# Recognition of a Fungal Effector Potentiates Pathogen‐Associated Molecular Pattern‐Triggered Immunity in Cotton

**DOI:** 10.1002/advs.202407787

**Published:** 2024-11-03

**Authors:** Lifan Sun, Xiangguo Li, Jiajie Zhong, Yu Wang, Baiyang Li, Ziqin Ye, Jie Zhang

**Affiliations:** ^1^ Institute of Microbiology Chinese Academy of Sciences Beijing 100101 China; ^2^ University of Chinese Academy of Sciences Beijing 100049 China

**Keywords:** cell death, effector, ETI, PAMP, plant immunity, PTI

## Abstract

Plants are equipped with multi‐layered immune systems that recognize pathogen‐derived elicitors to activate immunity. *Verticillium dahliae* is a soil‐borne fungus that infects a broad range of plants and causes devastating wilt disease. The mechanisms underlying immune recognition between plants and *V. dahliae* remain elusive. Here, a *V. dahliae* secretory protein, elicitor of plant defense gene (*VdEPD1*), acts as an elicitor that triggers defense responses in both *Nicotiana benthamiana* and cotton plants is identified. Targeted gene deletion of VdEPD1 enhances *V. dahliae* virulence in plants. Expression of *VdEPD1* triggers the accumulation of reactive oxygen species (ROS) and the activation of cell death in cotton plants. *Gossypium barbadense* EPD1‐interacting receptor‐like cytoplasmic kinase (GbEIR5A) and GbEIR5D interact with VdEPD1. Silencing of *GbEIR5A/D* significantly impairs VdEPD1‐triggered cell death in cotton plants, indicating the contribution of GbEIR5A/D to VdEPD1‐activated effector‐triggered immunity (ETI). VdEPD1 stimulates the expression of *GbEIR5A* and *GbEIR5D* in cotton plants. Interestingly, cotton plants with silenced *GbEIR5A/D* genes exhibit compromised pathogen‐associated molecular patterns (PAMPs)‐triggered ROS accumulation, whereas overexpression of GbEIR5A or GbEIR5D enhances PAMP‐induced ROS. These findings indicate that recognition of VdEPD1 potentiates GbEIRs to enhance cotton PAMP‐triggered immunity (PTI), uncovering a cooperative interplay of PTI and ETI in cotton.

## Graphical abstract

Plants have evolved sophisticated immune systems to defend against pathogens. Recent studies have indicated mutually promotive interactions between pathogen‐associated molecular pattern‐triggered immunity (PTI) and effector‐triggered immunity (ETI) in *Arabidopsis*. Here, it is shown that recognition of a fungal effector induces ETI and enhances PTI in cotton, suggesting a conserved cooperation between PTI and ETI.

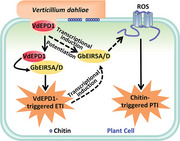

## 1 Introduction

Plants are equipped with sophisticated surveillance mechanisms to restrict the invasion of pathogenic microbes. Pathogen‐associated molecular patterns (PAMPs) are pathogen‐derived conserved molecules recognized by plants as foreign signatures, able to activate PAMP‐triggered immunity (PTI).^[^ ^1^ ^]^ Many of the successful pathogens secrete effectors to promote virulence. However, a few of these effectors are recognized by plant intracellular receptors such as nucleotide‐binding domain (NBD) and leucine‐rich repeat (LRR) receptors (collectively known as NLRs) to initiate effector‐triggered immunity (ETI). ETI is responsible for the triggering of the effector‐specific cell death known as hypersensitive response and the localized accumulation of reactive oxygen species (ROS), thereby acting as avirulence proteins.^[^ ^2^ ^]^ Recent studies have shown that certain components are crucial for both PTI and ETI. Furthermore, PTI and ETI potentiate each other, leading to stronger defense responses in the model plant *Arabidopsis thaliana*.^[^ ^3^, ^4^, ^5^, ^6^ ^]^ Advanced knowledge on PTI and ETI coevolution and cooperation has been established recently in *Arabidopsis*, whether this new paradiagram applies to crops, for instance cotton, remains unexplored. Cotton, being a significant industrial and economic crop, is extensively cultivated across the globe.^[^ ^7^ ^]^ However, the cotton immune mechanisms are less elucidated, and the PTI and ETI signaling frame in cotton have not been established.

The soil‐borne fungus *Verticillium dahliae* infects a broad range of plants, including cotton, secreting virulence factors that promote colonization and cause vascular wilt disease.^[^ ^8^, ^9^, ^10^, ^11^, ^12^, ^13^, ^14^ ^]^ Effectors are a subset of molecules mediating plant‐microbe interactions. They possess the ability to manipulate plant immunity and, under certain conditions, can be recognized by plants to elicit an immune response. Some effectors derived from *V. dahliae* are characterized as elicitors that activate plant immunity. Ave1 (Avirulence on Ve1 tomato) activates Ve1‐mediated resistance in plants carrying *Ve1*;^[^ ^15^ ^]^ Glycoside hydrolase 12 (GH12) protein endoglucanase 1 (VdEG1) and VdEG3 act as PAMP elicitors, as they trigger PTI immune responses in tomato, tobacco, and cotton plants.^[^ ^16^ ^]^ Ribotoxin 1 (VdRTX1), candidate effector 11 (VdCE11), Vd424Y, and secreted protein 3 （SP3) have also been found to carry elicitor activity.^[^ ^17^, ^18^, ^19^, ^20^ ^]^ Toxic factors secreted by *V. dahliae*, xylanase 4 (VdXyn4), Nep1‐like proteins (VdNLP1), and VdNLP2 have been reported to play roles in disease development, causing foliar wilting via cell wall degradation, host response manipulation, or cytotoxicity.^[^ ^21^, ^22^, ^23^, ^24^, ^25^, ^26^, ^27^ ^]^

Plant receptor‐like cytoplasmic kinases (RLCKs) belong to a protein kinase family that regulates signal transduction, overseeing a wide range of biological processes.^[^ ^28^, ^29^, ^30^, ^31^ ^]^ Botrytis‐induced kinase1 (BIK1) and its closely related kinase PBS1‐like1 (PBL1) from the *Arabidopsis* RLCK subfamily VII play crucial roles downstream of multiple pattern‐recognition receptors (PRRs), including Flagellin sensing 2 (FLS2), EF‐Tu receptor (EFR), chitin elicitor receptor kinase 1 (CERK1), and plant elicitor peptide (Pep) receptors 1/2 (PEPR1/2).^[^ ^32^, ^33^, ^34^, ^35^, ^36^, ^37^ ^]^ The *Arabidopsis* RPM1‐induced protein kinase (RIPK), a subfamily VII member, is required for ETI triggered by the *Pseudomonas syringae* effector AvrB.^[^ ^38^, ^39^, ^40^ ^]^ The modulation of RLCKs by a microbial effector during PTI suppression and ETI activation suggests their role in mediating the evolutionary links between PTI and ETI. Although research on RLCKs has made some progress in *Arabidopsis* and rice, research in cotton is still in its infancy. Phylogenetic analysis, possibly using *Arabidopsis* as a reference, identified ≈100 RLCK‐VII members in the *Gossypium hirsutum* genome, grouped into nine distinct categories,^[^ ^41^ ^]^ whose roles in cotton immunity remain largely unexplored.

In this study, we have identified a new pair of recognition proteins in the *V. dahliae*‐cotton interaction. Elicitor of plant defense gene (VdEPD1) acts as a secretory protein in *V. dahliae* that exhibits elicitor activity. Cotton plants exhibited reduced resistance to the Vd∆*epd1* mutant compared with the *V. dahliae* wildtype (WT) strain, indicating that VdEPD1 is involved in pathogen recognition and cotton resistance to *V. dahliae*. *Gossypium barbadense* EPD1‐interacting receptor‐like cytoplasmic kinase (GbEIR5A) and GbEIR5D are involved in VdEPD1‐activated ETI. VdEPD1 potentiates GbEIR5A and GbEIR5D to enhance cotton PTI. These findings identified a node of PTI‐ETI crosstalk in cotton, indicating the potentiation of PTI by ETI in cotton.

## 2 Result

### 2.1 A T‐DNA Insertional Mutant of *V. dahliae* Exhibits Enhanced Pathogenicity in Cotton Plants

**Figure 1:**
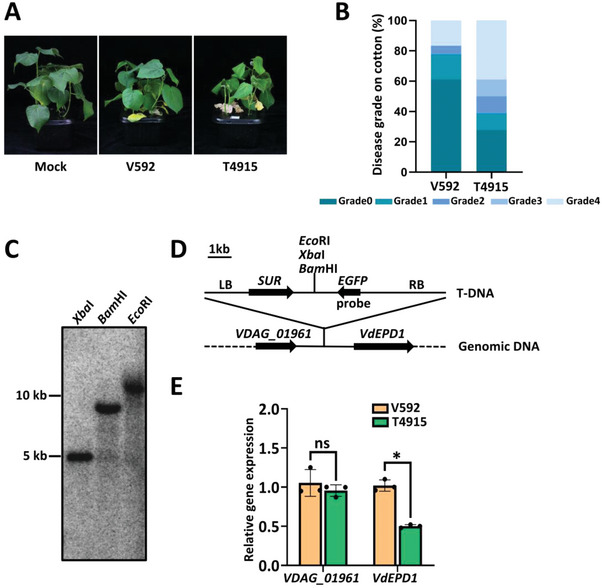
A T‐DNA insertional *V. dahliae* mutant exhibits enhanced pathogenicity in cotton plants. A,B), Disease symptoms **A**) and index analyses **B**) of cotton infected with V592 or the T4915 mutant strain. Three‐week‐old cotton seedlings were inoculated with V592 or T4915. The plants were photographed and subjected to disease index analyses 4–5 weeks post‐inoculation. Disease indexes were evaluated with three replicates generated from 18 plants (*n* = 18) for each inoculum. C) Southern blot analysis indicates a single copy of T‐DNA insertion in the T4915 mutant. The genomic DNA of T4915 was digested with *Xba*I, *Bam*HI, or *Eco*RI respectively, and used for Southern blot analysis with a probe corresponding to GFP coding sequence. D) Schematic description of the T‐DNA insertion site in the T4915 mutant. E) Expression of *VdEPD1* is downregulated in the T4915 mutant. The conidia of V592 and T4915 mutants were cultured in a PDB medium for 3 days. Total RNA was extracted and used to determine the transcriptional levels of *VDAG_01961* and *VdEPD1*. *VdELF1* was used as an internal control. Values are the means ± SD; *n* = 3. Error bars indicate the standard deviation of three biological replicates. The experiments were repeated three times with similar results. Student's *t*‐test was carried out to determine the significance of the difference. ^*^ indicates a significant difference at a *p*‐value of <0.05. ns indicates no significant difference.

To identify *V. dahliae* components recognized by plants and able to trigger immunity, we performed a genetic screening to isolate *V. dahliae* mutants exhibiting enhanced pathogenicity in cotton (*Gossypium barbadense*) plants, which exhibit higher resistance than *G. hirsutum* to *V. dahliae* (Figure S1A,B, Supporting Information). For this purpose, we used our T‐DNA insertional mutant library, previously constructed^[^ ^42^ ^]^ for screening. A T‐DNA insertional mutant (T4915) exhibiting more severe disease symptoms in *G. barbadense* plants than the WT strain V592 was isolated^[^ ^26^, ^42^ ^]^ (**Figure** **1**A,B). To identify the mutations responsible for the enhanced pathogenicity, we next characterized the T‐DNA insertion sites in the T4915 mutant. Southern blot analysis indicated a single copy of the T‐DNA integrated into the V592 genomic region (Figure 1C) between *VDAG_01961* and *VDAG_01962* (*VdEPD1)* coding sequences (Figure 1D). We next examined the expression levels of *VDAG_01961* and *VdEPD1* in the T4915 mutant and the WT strain V592 and observed downregulation of *VdEPD1*, but not of *VDAG_01961*, in the T4915 mutant compared to the V592 strain (Figure 1E).

### 2.2 Deletion of *VdEPD1* Enhances the Pathogenicity of *V. dahliae*

**Figure 2:**
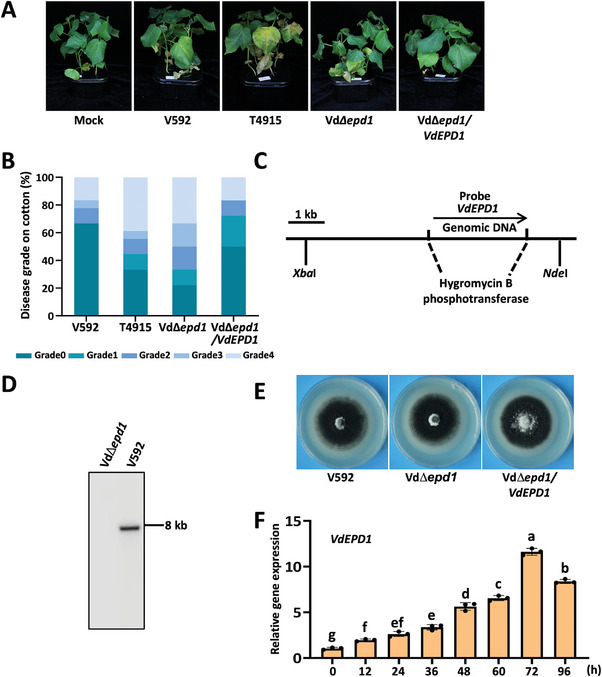
Generation and colony morphology of the Vd∆*epd1* mutant, which exhibits enhanced virulence in cotton plants. A,B), Disease symptoms A) and index analyses B) of cotton infected with V592, T4915, VdΔ*epd1*, or VdΔ*epd1/VdEPD1* strains. Three‐week‐old cotton seedlings were inoculated by V592, T4915, VdΔ*epd1*, or VdΔ*epd1*/*VdEPD1*. The plants were photographed and subjected to disease index analyses 4–5 weeks post‐inoculation. Disease indexes were evaluated with three replicates generated from 18 plants (*n* = 18) for each inoculum. C), Schematic description of *VDAG_01962* (*VdEPD1*) gene deletion. D), Southern blot analysis indicates *VdEPD1* gene deletion in the VdΔ*epd1* mutant. Genomic DNA samples isolated from V592 and VdΔ*epd1* strains were digested with *Xba*I and *Nde*I and used for Southern blot analysis. E), Morphology of V592, VdΔ*epd1*, and VdΔ*epd1/VdEPD1* strains grown on PDA plates. The colonies of the indicated strains were photographed 2 weeks post‐inoculation. F), *VdEPD1* is upregulated at an early stage of cotton colonization. The expression level of *VdEPD1* in V592 conidia or at 12, 24, 36, 48, 60, 72, 96 hours post‐inoculation with cotton was detected. Total RNA was extracted from cotton roots infected by V592 for RT‐qPCR analyses of *VdEPD1* expression. Values are the means ± SD; *n* = 3. Error bars indicate the standard deviation of three biological replicates. Different letters indicate significant differences, as determined by one‐way ANOVA.

To investigate whether the enhanced pathogenicity of T4915 resulted from *VdEPD1* downregulation, two targeted gene deletion mutants, Vd*∆01962* (deletion of *VDAG_01962*, designated as Vd*∆epd1*) and Vd*∆01961* (deletion of *VDAG_01961*), were subsequently generated by homologous recombination^[^ ^43^ ^]^ and examined for pathogenicity in *G. barbadense* plants. The Vd*∆epd1* mutant, but not the Vd*∆01961* mutant, displayed enhanced virulence compared with the V592 (**Figure** **2A,B**; Figure S1C,D, Supporting Information). Deletions of *VdEPD1* and *VDAG_01961* in the Vd*∆epd1* and Vd*∆01961* mutants, respectively, were confirmed by Southern blot analysis (Figure 2C,D; Figure S1E,F, Supporting Information). The Vd*∆epd1* deletion mutant displayed normal growth on PDA plates (Figure 2E). Furthermore, the enhanced virulence of the Vd*∆epd1* mutant was suppressed upon complementation with the genomic coding region of *VdEPD1* (Vd*∆epd1/VdEPD1*) (Figure 2A,B).

To verify whether the enhanced pathogenicity of the T4915 mutant was associated with *VdEPD1* downregulation, the genomic coding region of *VdEPD1* was subsequently introduced into the T4915 mutant to generate a new strain, T4915/*VdEPD1*. This strain exhibited comparable pathogenicity to the WT strain V592 (Figure S2A,B, Supporting Information), suggesting that, as hypothesized, *VdEPD1* downregulation caused the increased pathogenicity of the T4915 mutant. We then examined the expression of *VdEPD1* at different stages during the early infection process. In support of a putative function for *VdEPD1* in plant‐*V. dahliae* interaction, gene expression analyses revealed that *VdEPD1* was significantly upregulated 12 hours after the beginning of the incubation period with cotton roots, reaching a peak at 72 hours (Figure 2F). These results suggest a causal relationship between *VdEPD1* downregulation and the enhanced pathogenicity of the T4915 mutant.

### 2.3 VdEPD1 Acts as an Effector and Elicits Cell Death and Defense Responses in Plants

**Figure 3:**
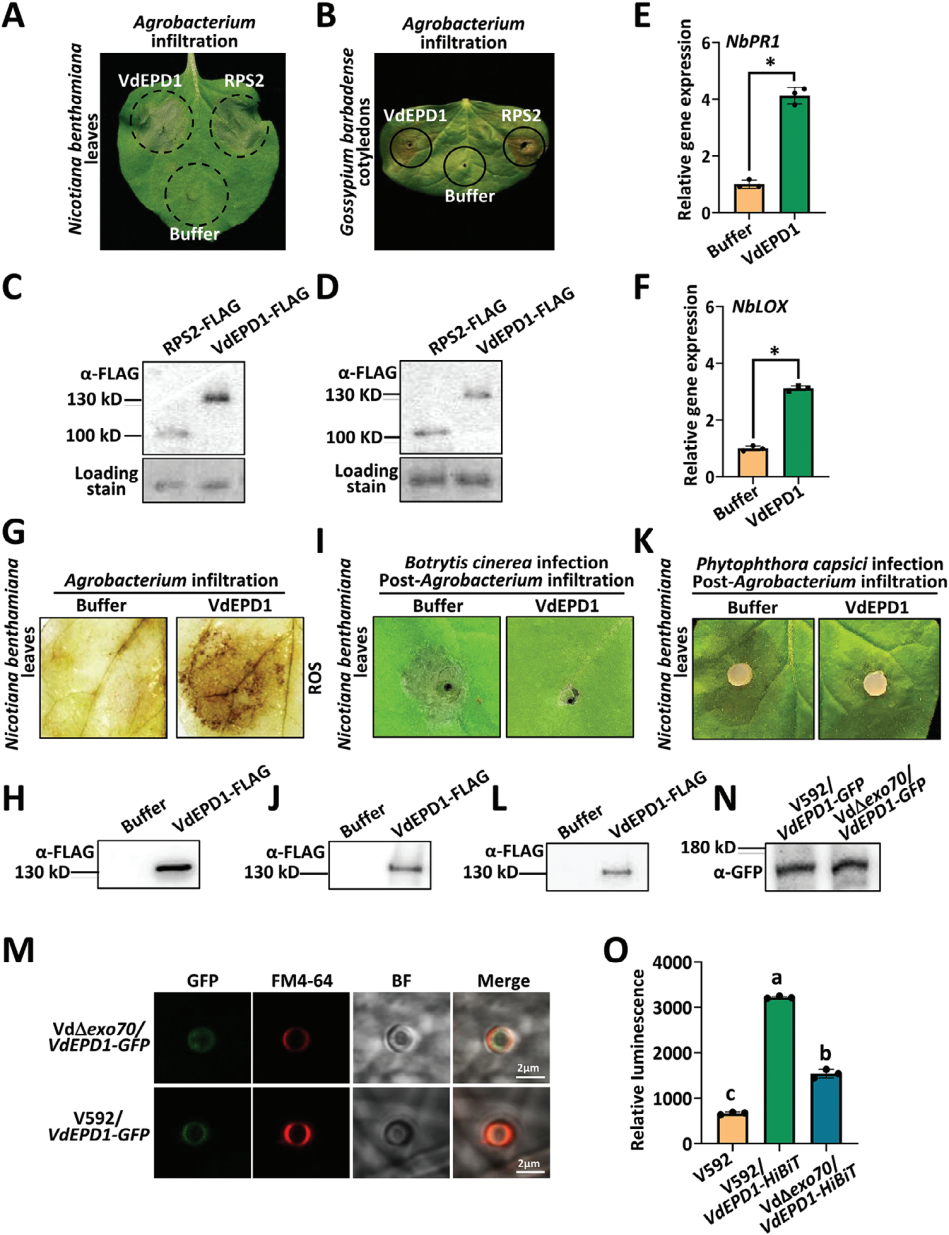
VdEPD1 acts as an effector that elicits cell death and defense responses in plants. A,B) Transient expression of VdEPD1 induced cell death in *N. benthamiana* A) and *G*. *barbadense* B). The *Agrobacterium* carrying *pCambia1300‐35S‐VdEPD1‐FLAG* (OD_600_: 0.8) was infiltrated into *N. benthamiana* leaves A) or *G*. *barbadense* leaves B). C,D) Expression levels of RPS2‐FLAG and VdEPD1‐FLAG proteins in *N. benthamiana* C) and *G*. *barbadense* D). *N. benthamiana* or *G*. *barbadense* leaves were infiltrated with the *Agrobacterium* strain as indicated. Total protein was extracted for anti‐FLAG western blotting. E,F), Transient expression of VdEPD1 induced the expression of defense‐related genes in *N. benthamiana. N. benthamiana* leaves were infiltrated with *Agrobacterium* carrying *pCambia1300‐35S‐VdEPD1‐FLAG* (OD_600_: 0.8). Total RNA from infiltrated plants was extracted for RT‐qPCR analyses of *NbPR1* E) and *NbLOX* F) expression at 2 days post‐inoculation. Values are the means ± SD; *n* = 3. Error bars indicate the standard deviation of three biological replicates. Student's *t*‐test was carried out to determine the significance of the difference. * indicates a significant difference at a *p*‐value of <0.05. G) Transient expression of VdEPD1 induced the accumulation of ROS in *N. benthamiana*. The leaves of *N. benthamiana* were stained with DAB at 48 hours post‐inoculation with *Agrobacterium* carrying *pCambia1300‐35S‐VdEPD1‐FLAG* (OD_600_: 0.8). H) Expression level of VdEPD1‐FLAG protein in *N. benthamiana*. *N. benthamiana* leaves were infiltrated with *Agrobacterium* strain as indicated. Total protein was extracted for anti‐FLAG western blotting. I) Transient expression of VdEPD1 enhanced plant resistance against *B. cinerea*. 10^6^/mL spores of *B. cinerea* were inoculated onto sites on leaves that had been infiltrated with *Agrobacterium* carrying *pCambia1300‐35S‐VdEPD1‐FLAG* (OD_600_: 0.08) 1 day previously. J) the Expression level of VdEPD1‐FLAG protein in *N. benthamiana*. *N. benthamiana* leaves were infiltrated with *Agrobacterium* strain as indicated and then inoculated with *B. cinerea*. Total protein was extracted for anti‐FLAG western blotting. K) Transient expression of VdEPD1 enhanced plant resistance against *P. capsici*. A 5 mm diameter of *P. capsici* mycelial plug was inoculated onto sites on leaves that had been infiltrated with *Agrobacterium* carrying *pCambia1300‐35S‐VdEPD1‐FLAG* (OD_600_: 0.08) 1 day previously. L) Expression level of VdEPD1‐FLAG protein in *N. benthamiana*. *N. benthamiana* leaves were infiltrated with *Agrobacterium* strain as indicated and then inoculated with *P. capsici*. Total protein was extracted for anti‐FLAG western blotting. M) VdEPD1 localizes at the base of the hyphopodium and forms ring signals surrounding the hyphal neck. A significant portion of VdEPD1‐GFP is sequestered within the hyphopodium in the Vd*∆exo70* mutant strain. The Vd*∆exo70*/*VdEPD1‐GFP* and V592/*VdEPD1‐GFP* strains were cultured on a cellophane membrane for 3 days to induce the formation of hyphopodia. Localization of VdEPD1‐GFP was visualized using a Leica SP8 microscope. N) Expression level of VdEPD1‐GFP in V592 and Vd∆*exo70*. The total protein of V592/*VdEPD1‐GFP* and Vd∆*exo70*/*VdEPD1‐GFP* was extracted for anti‐GFP western blotting. O) VdEPD1‐HiBiT is secreted into the culture medium. The Vd∆*exo70*/*VdEPD1‐HiBiT*, V592/*VdEPD1‐HiBiT*, and V592 strains were cultured in a PDB medium and incubated with cotton roots. The culture media were collected and incubated with NlucN‐His and 10 µm coelenterazine. The presence of VdEPD1‐HiBiT was detected by a microplate luminometer. Values are the means ± SD; *n* = 3. Error bars indicate the standard deviation of three biological replicates. The experiments were repeated 3 times with similar results. Different letters indicate significant differences, as determined by one‐way ANOVA.

The elevated pathogenicity of the Vd*∆epd1* mutant prompted us to test the putative immune elicitor activity of VdEPD1. For this purpose, *VdEPD1* was next transiently expressed in *Nicotiana benthamiana* leaves via *Agrobacterium*‐mediated transformation. As expected, the transient expression of VdEPD1 elicited cell death in *N. benthamiana* (**Figure** **3**A). The *Arabidopsis* NLR protein RPS2 (resistant to *Pseudomonas syringae* 2) was used as a control to elicit cell death in *N. benthamiana*.^[^ ^44^ ^]^ Similarly, the transient expression of VdEPD1 also elicited cell death in *G. barbadense* and *G. hirsutum* leaves (Figure 3B; Figure S3A, Supporting Information). The expression of VdEPD1 and RPS2 proteins in *N. benthamiana* (Figure 3C) and *G. barbadense* (Figure 3D), as well as the expression of VdEPD1 in *G. hirsutum* (Figure S3B, Supporting Information), was confirmed by immunoblotting. To determine the core domain responsible for eliciting cell death in VdEPD1, we constructed the vectors carrying different segments (VdEPD1‐1, VdEPD1‐2, VdEPD1‐3, and VdEPD1‐4) (Figure S3C, Supporting Information). Subsequently, these proteins were expressed in *N. benthamiana*. The full length of VdEPD1, but not other segments, can trigger cell death in *N. benthamiana*, indicating that the full‐length protein is required for VdEPD1 to induce cell death in *N. benthamiana* (Figure S3D, Supporting Information). The expression of VdEPD1‐1, VdEPD1‐2, VdEPD1‐3, VdEPD1‐4, and VdEPD1 proteins was confirmed by immunoblotting (Figure S3E,F, Supporting Information). These results demonstrate an active immune elicitor activity of the full‐length VdEPD1 protein. A BLAST search revealed a wide distribution of VdEPD1 homologous proteins in many fungi with high sequence similarity (Figure S4, Supporting Information). We further cloned VdEPD1 homologs from *Fusarium oxysporum* (FoEPD1) and *Fusarium graminearum* (FgEPD1). The expression of either of these two VdEPD1 homologs induced cell death in *N. benthamiana* plants (Figure S5A,B, Supporting Information). The expression of FoEPD1 and FgEPD1 proteins was confirmed by immunoblotting (Figure S5C,D, Supporting Information). The results indicate a conservative elicitor activity of VdEPD1 homologs in plants, suggesting that VdEPD1‐mediated recognition may also occur in other plant‐fungus interactions.

We further examined the expression of defense‐related genes and the accumulation of ROS following the transient expression of *VdEPD1*. The expression of the defense‐related genes *NbPR1* and *NbLOX* was induced in *N. benthamiana* plants expressing *VdEPD1* (Figure 3E,F), and ROS accumulation occurred (Figure 3G). The expression of VdEPD1 proteins was confirmed by immunoblotting (Figure 3H). To further verify the elicitor activity of VdEPD1, the broad‐host‐range filamentous pathogen *Botrytis cinerea* and the semibiotrophic pathogen *Phytophthora capsici* were inoculated onto control and VdEPD1‐expressing *N. benthamiana* leaves. *VdEPD1*‐expressing leaves exhibited significantly enhanced resistance to both *B. cinerea* (Figure 3I,J) and *P. capsici* (Figure 3K,L) compared to control leaves. Taken together, the above results indicate that VdEPD1 elicits immune responses in plants.

Many elicitors triggering plant NLR‐mediated immunity are secretory proteins. In *V. dahliae*, the septin‐ring‐organized hyphal neck acts as a functional fungus‐host penetration machinery to deliver secretory proteins. This process usually requires the formation of an exocyst complex for efficient secretion.^[^ ^14^ ^]^ Based on these concepts, we further examined the localization of VdEPD1. VdEPD1‐GFP was introduced into the WT strain V592 and VdΔ*exo70* mutant (unable to form the exocyst complex) and incubated on cellophane for hyphopodium induction. The GFP‐imaging assay demonstrated the localization of VdEPD1 at the hyphal neck in the V592 strain; however, a great portion of VdEPD1‐GFP was sequestered within the hyphopodium in the VdΔ*exo70* mutant strain (Figure 3M). The expression of the VdEPD1 protein was confirmed by immunoblotting (Figure 3N). To further examine whether VdEPD1 is a secretory protein, a self‐assembling split Nano luciferase (NanoLuc)‐based assay was utilized for detecting VdEPD1 secretion.^[^ ^45^ ^]^ VdEPD1 fused with a smaller C‐terminal fragment of NanoLuc (HiBiT) was introduced into the WT strain V592 and VdΔ*exo70* mutant. The supernatants of culture media of V592, V592/*VdEPD1‐HiBiT*, and VdΔ*exo70*/*VdEPD1‐HiBiT* strains were collected and incubated with the recombinant protein of a larger N‐terminal fragment of NanoLuc (NanoLucN‐His) and coelenterazine, then the luminescence was monitored. The HiBiT values for V592/VdEPD1‐HiBiT are significantly elevated compared to those of V592 and VdΔ*exo70*/VdEPD1‐HiBiT, suggesting that VdEPD1‐HiBiT is secreted and subsequently binds to NanoLucN (Figure 3O). We further examined the putative translocation of *V. dahliae*‐delivered VdEPD1 into the plant cells. The V592 strains expressing VdEPD1‐GFP (V592/*VdEPD1‐GFP*) and GFP (V592/*GFP*) were separately inoculated onto onion epidermal cells. Although GFP fluorescence from the V592/GFP strain was observed in conidial spores, VdEPD1‐GFP secreted by *V. dahliae* was capable of translocating into plant cells (Figure S6, Supporting Information). The results suggest that VdEPD1 is a secretory protein that is delivered by *V. dahliae* and translocates into the plant cell.

### 2.4 VdEPD1‐Induced Cell Death in *N. Benthamiana* Requires *NbS00058880g0009*

**Figure 4:**
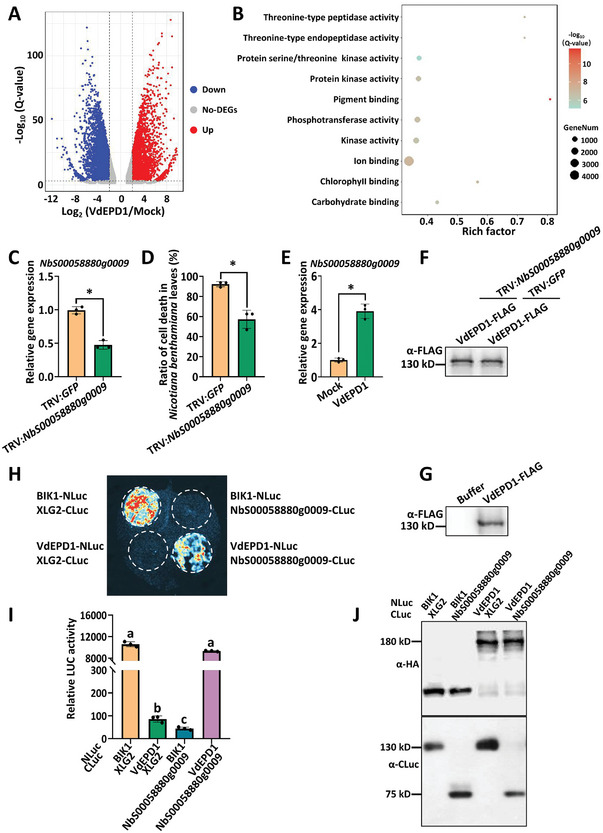
NbS00058880g0009 binds to VdEPD1 and is required for VdEPD1‐induced cell death in *N. benthamiana*. A) Volcano plots were generated to illustrate differentially expressed VdEPD1‐responsive genes in *N. benthamiana* plants. Leaves from four‐week‐old *N. benthamiana* plants were infiltrated with buffer or *Agrobacterium* carrying *pCambia1300‐35S‐VdEPD1‐FLAG* (OD_600_: 0.8). Total RNA was extracted 34 hours post‐inoculation with *Agrobacterium* from the infiltrated plants for RNA sequencing analyses. Shown in *x*‐axis are log_2_‐fold changes of transcripts in VdEPD1 compared with Mock. A significant change (fold change >2, *p* < 0.05) appears in blue (downregulated genes) or red (upregulated genes), and gray means no differentially expressed genes (DEGs). B) GO enrichment analysis of genes significantly up‐regulated and down‐regulated in response to VdEPD1 expression. The top ten enriched terms in the gene set are listed. C) The expression level of NbS00058880g0009 is reduced in *N. benthamiana* plants that have been infiltrated with *Agrobacterium* carrying *pTRV1* and *pTRV2‐NbS00058880g0009*. Total RNA was extracted from the infiltrated plants for RT‐qPCR analysis of *pTRV2‐NbS00058880g0009* expression at 3–4 weeks post‐inoculation. D) Silencing of *NbS00058880g0009* compromises VdEPD1‐induced cell death. *Agrobacterium* carrying *pCambia1300‐35S‐VdEPD1‐FLAG* construct was infiltrated into *NbS00058880g0009*‐silenced or *GFP*‐silenced *N. benthamiana* plants. The percentage of cell death was recorded at 3 days post‐inoculation. E) The expression level of *NbS00058880g0009* is induced in plants infiltrated with *Agrobacterium* carrying *pCambia1300‐35S‐VdEPD1‐FLAG*. Total RNA was extracted from the infiltrated plants for RT‐qPCR analysis of *NbS00058880g0009* expression at 34 hours post‐inoculation, with *NbACTIN* serving as an internal control. Values are the means ± SD; *n* = 3 C–E). Error bars indicate the standard deviation of three biological replicates. Student's *t*‐test was carried out to determine the significance of the difference. ^*^ indicates a significant difference at a *p*‐value of <0.05. F,G) Expression levels of VdEPD1‐FLAG protein in *N. benthamiana* used for VdEPD1‐induced cell death F) and inducing expression of *NbS00058880g0009* G). *N. benthamiana* leaves were infiltrated with *Agrobacterium* strain as indicated. Total protein was extracted for anti‐FLAG western blotting. H) VdEPD1 interacts with NbS00058880g0009 in *N. benthamiana*. Luciferase imaging was used to visualize the interaction between VdEPD1 and NbS00058880g0009 in *N. benthamiana* leaves. *N. benthamiana* leaves infiltrated with *Agrobacterium* carrying the indicated constructs were subjected to a luciferase complementation imaging assay. XLG2, a GTP‐binding protein in *Arabidopsis* not expected to interact with VdEPD1, was used to present the basal level of the signal associated with the split luciferase assay itself. I) Quantitative luminescence of VdEPD1 and NbS00058880g0009 interaction. *N. benthamiana* leaves infiltrated with the indicated constructs were sliced into strips, and their relative luminescence was determined using a microplate luminometer. Values are the means ± SD; *n* = 3. Error bars indicate the standard deviation of 3 biological replicates. Different letters indicate significant differences, as determined by one‐way ANOVA. J) All genes encoding NLuc proteins were further fused with a 3×HA tag in the NLuc‐vector. Anti‐HA and anti‐CLuc immunoblots were used to detect the levels of NLuc‐fusion and CLuc‐fusion proteins, respectively.

To explore the components that are responsible for the recognition of VdEPD1 in *N. benthamiana*, we performed transcriptome sequencing on *N. benthamiana* leaves that were infiltrated with buffer (designated as Mock) and *Agrobacterium* carrying *pCambia1300‐35S‐VdEPD1‐FLAG* (designated as VdEPD1). Pearson correlation coefficient matrix quantifies the similarity and correlation between the transcriptome samples (Figure S7A, Supporting Information). Principal Component Analysis visualizes the variation and clustering patterns among transcriptome samples based on their expression values measured in Fragments Per Kilobase of transcript per Million mapped reads (Figure S7B, Supporting Information). We defined genes with twofold increase or decrease in VdEPD1 samples compared with Mock as VdEPD1‐responsive genes (**Figure** **4**A). Gene Ontology (GO) enrichment analysis of the regulated genes revealed that three of the top ten terms were related to protein kinase genes, indicating that these genes play crucial roles in plant responses to VdEPD1 (Figure 4B). To investigate whether VdEPD1‐triggered cell death requires any of the protein kinase‐related genes, twenty‐seven protein‐related kinases that are upregulated 10‐fold in the transcription of VdEPD1 samples were screened out for further analyses. *Agrobacterium* harboring a virus‐induced gene silencing (VIGS) vector for each of these genes was infiltrated with *pTRV1* into *N. benthamiana* plants individually. Subsequently, VdEPD1 protein was expressed in the leaves of *N. benthamiana* with the protein‐related kinase genes silenced. The reduced expression of the protein‐related kinase genes in plants was verified using real‐time quantitative PCR (RT‐qPCR). (Figure 4C; Figure S8A, Supporting Information). The silencing of *NbS00058880g0009*, but not of most other genes, compromised VdEPD1‐induced cell death significantly (Figure 4D; Figure S8B, Supporting Information), and *NbS00058880g0009* is significantly upregulated by VdEPD1 (Figure 4E). The expression of VdEPD1 protein in *N. benthamiana* was confirmed by immunoblotting (Figure 4F,G).

*NbS00058880g0009* encodes a putative receptor‐like protein kinase in *N. benthamiana* (Figure S9, Supporting Information). Previous studies have demonstrated that some RLCKs in *Arabidopsis* are directly targeted by bacterial effectors, for example, the *Pseudomonas syringae* effector AvrB and HopZ1a, and the *Xanthomonas campestris* effector AvrAC.^[^ ^39^, ^40^, ^46^, ^47^, ^48^, ^49^, ^50^, ^51^, ^52^ ^]^ These examples prompted us to examine whether VdEPD1 associates with NbS00058880g0009. A split luciferase assay was then conducted to test a putative interaction between NbS00058880g0009 and VdEPD1. Co‐expression of the N terminus of luciferase (NLuc)‐tagged VdEPD1 and the C terminus of luciferase (CLuc)‐tagged NbS00058880g0009 resulted in much higher luciferase activity compared to the negative control (Figure 4H,I), thereby indicating a specific interaction between NbS00058880g0009, designated as NbEIR (EPD1‐interacting receptor‐like cytoplasmic kinase), and VdEPD1. The expression levels of the NLuc‐HA and CLuc‐fusion proteins were further detected by immunoblotting (Figure 4J). These results suggest that VdEPD1 is likely recognized by plants to trigger immune responses that require NbEIR.

### 2.5 GbEIR5A/D Are Required for Resistance to *V. dahliae* in *G. barbadense*

**Figure 5:**
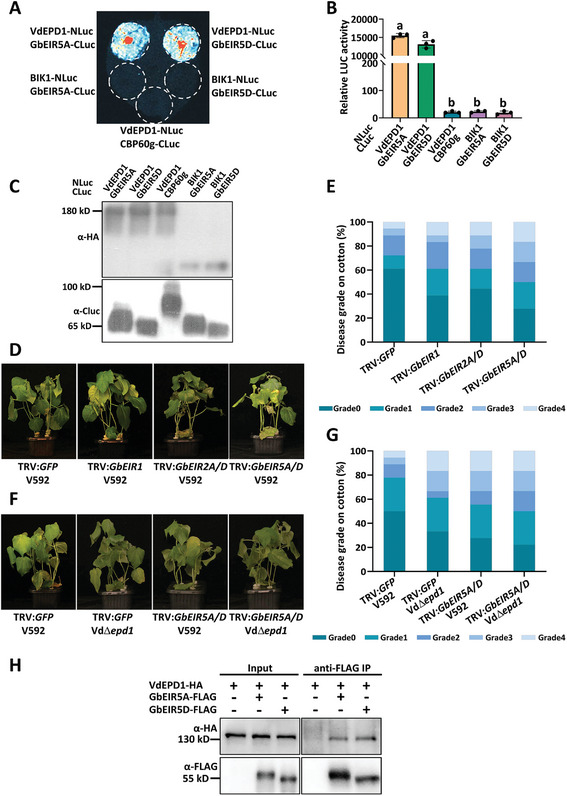
GbEIR5A/D bind to VdEPD1 and contributes to VdEPD1‐triggered defense. A) VdEPD1 interacts with GbEIR5A/D in *N. benthamiana*. Luciferase imaging of VdEPD1 and GbEIR5A/D interaction in *N. benthamiana* leaves. *N. benthamiana* leaves infiltrated with *Agrobacterium* carrying the indicated constructs were subjected to a luciferase complementation imaging assay. B) Quantitative luminescence of VdEPD1 and GbEIR5A/D interaction. *N. benthamiana* leaves infiltrated with the indicated constructs were sliced into strips, and their relative luminescence was determined using a microplate luminometer. Values are the means ± SD; *n* = 3. Error bars indicate the standard deviation of 3 biological replicates. Different letters indicate significant differences, as determined by one‐way ANOVA. C) All genes encoding NLuc proteins were further fused with a 3×HA tag in the NLuc‐vector. Anti‐HA and anti‐CLuc immunoblots were used to detect the levels of NLuc‐fusion and CLuc‐fusion proteins, respectively. D,E) Silencing of *GbEIR5A/D* compromised resistance to *V. dahliae*. Disease symptoms D) and index analyses E) of cotton infiltrated with *Agrobacterium* carrying *pTRV1* together with *pTRV2‐GFP*, *pTRV2‐GbEIR1*, *pTRV2‐GbEIR2A*/*D* or *pTRV2‐GbEIR5A/D* as indicated. *V. dahliae* strain V592 was inoculated 10 days post *Agrobacterium* infiltration. The plants were photographed and subjected to disease index analyses 4–5 weeks post‐inoculation. Disease indexes were evaluated with 3 replicates generated from 18 plants (*n* = 18) for each inoculum. F,G) GbEIR5A/D are required for VdEPD1‐mediated defense. Disease symptoms F) and index analyses G) of cotton infiltrated with *Agrobacterium* carrying *pTRV1* together with *pTRV2‐GFP*, *pTRV2‐GbEIR5A/D* as indicated. *V. dahliae* strain V592 and Vd∆*epd1* were inoculated 10 days post *Agrobacterium* infiltration. The plants were photographed and subjected to disease index analyses 4–5 weeks post‐inoculation. Disease indexes were evaluated with three replicates generated from 18 plants (*n* = 18) for each inoculum. H) VdEPD1 interacts with GbEIR5A/D in *Arabidopsis*. *Arabidopsis* protoplasts were transfected with VdEPD1‐HA alone or together with GbEIR5A/D‐FLAG as indicated. Protein was extracted 12 hours post‐transfection and immunoprecipitated with anti‐FLAG. Anti‐HA or anti‐FLAG immunoblot was used to detect the presence of VdEPD1‐HA or GbEIR5A/D‐FLAG in the purified complex, respectively.

To investigate the recognition mechanisms of VdEPD1 in cotton, homologous proteins of NbEIR in *G. barbadense* were screened out based on sequence similarity and designated as GbEIR1, GbEIR2A, GbEIR2D, GbEIR3A, GbEIR3D, GbEIR4, GbEIR5A, and GbEIR5D (Figure S10, Supporting Information). A split‐luciferase assay was subsequently conducted to test putative interactions between GbEIRs and VdEPD1. The co‐expression of VdEPD1 tagged with NLuc and GbEIRs tagged with CLuc resulted in significantly elevated luciferase activity in several GbEIRs (Figure S11A, Supporting Information). The result suggests that a few GbEIRs, including GbEIR1, GbEIR2A, GbEIR2D, GbEIR5A, and GbEIR5D, are able to interact with VdEPD1 (**Figure** **5**A,B; Figure S11A, Supporting Information). The expression levels of the NLuc‐HA and CLuc‐fusion proteins were further detected by immunoblotting (Figure 5C; Figure S11B, Supporting Information).

To investigate whether the five candidate genes contribute to plant resistance against *V. dahliae*, we constructed VIGS vectors targeting these genes and infiltrated them into cotton cotyledons to silence the candidate genes.^[^ ^53^, ^54^, ^55^, ^56^ ^]^ The G*bEIR5A* and G*bEIR5D* double silenced plants (*GbEIR5A/D*) exhibited enhanced disease symptoms (Figure 5D,E). The *Cloroplastos Alterados 1* gene (*CLA1*) was used as a control for efficient VIGS in *G. barbadense* ^[^ ^56^ ^]^ (Figure S12A, Supporting Information). The reduced expression of *GbEIR1*, *GbEIR2A*, *GbEIR2D*, *GbEIR5A*, or *GbEIR5D* in plants was verified using RT‐qPCR (Figure S12B–D, Supporting Information). These results suggest that GbEIR5A and GbEIR5D interact with VdEPD1, playing a crucial role in plant resistance against *V. dahliae*. We then investigated the requirement of GbEIR5A and GbEIR5D in VdEPD1‐mediated defense. The *GFP*‐silenced and *GbEIR5A/D*‐silenced cotton plants were inoculated with both *V. dahliae* WT strain V592 and the Vd∆*epd1* mutant. When compared with V592, the Vd∆*epd1* mutant displayed enhanced virulence in the *GFP*‐silenced plants. However, this enhanced virulence resulting from the deletion of VdEPD1 was partially impaired in the *GbEIR5A/D*‐silenced cotton plants, compared to the *GFP*‐silenced plants (Figure 5F,G). The results indicate that GbEIR5A and GbEIR5D are required for VdEPD1‐mediated defense.

The interaction between VdEPD1 and GbEIR5A or GbEIR5D was further verified by co‐immunoprecipitation (Co‐IP) analysis. HA‐tagged VdEPD1 was transfected, either alone or together with FLAG‐tagged GbEIR5A or GbEIR5D, into *Arabidopsis* protoplasts for transient expression. Anti‐FLAG IP followed by an anti‐HA immunoblot revealed that VdEPD1 was co‐purified with GbEIR5A or GbEIR5D from plant cells (Figure 5H). VdEPD1‐GFP and GbEIR5A‐mCherry were then co‐transiently expressed in both *G. barbadense* protoplasts and in *G. barbadense* leaves. GFP and mCherry fluorescence imaging revealed that VdEPD1 and GbEIR5A co‐localized in *G. barbadense* cells (Figure S13A,B, Supporting Information). A similar localization pattern was also observed in *N. benthamiana* (Figure S13C,D, Supporting Information). RT‐qPCR analysis was performed using total RNA extracted from roots and true leaves of *G. barbadense* to investigate the expression of *GbEIR5A/D*. The results showed that GbEIR5A/D had higher expression in the roots of *G. barbadense* than in true leaves. (Figure S14, Supporting Information). These results suggest that GbEIR5A and GbEIR5D interact with VdEPD1, playing a crucial role in plant resistance against *V. dahliae*.

**Figure 6:**
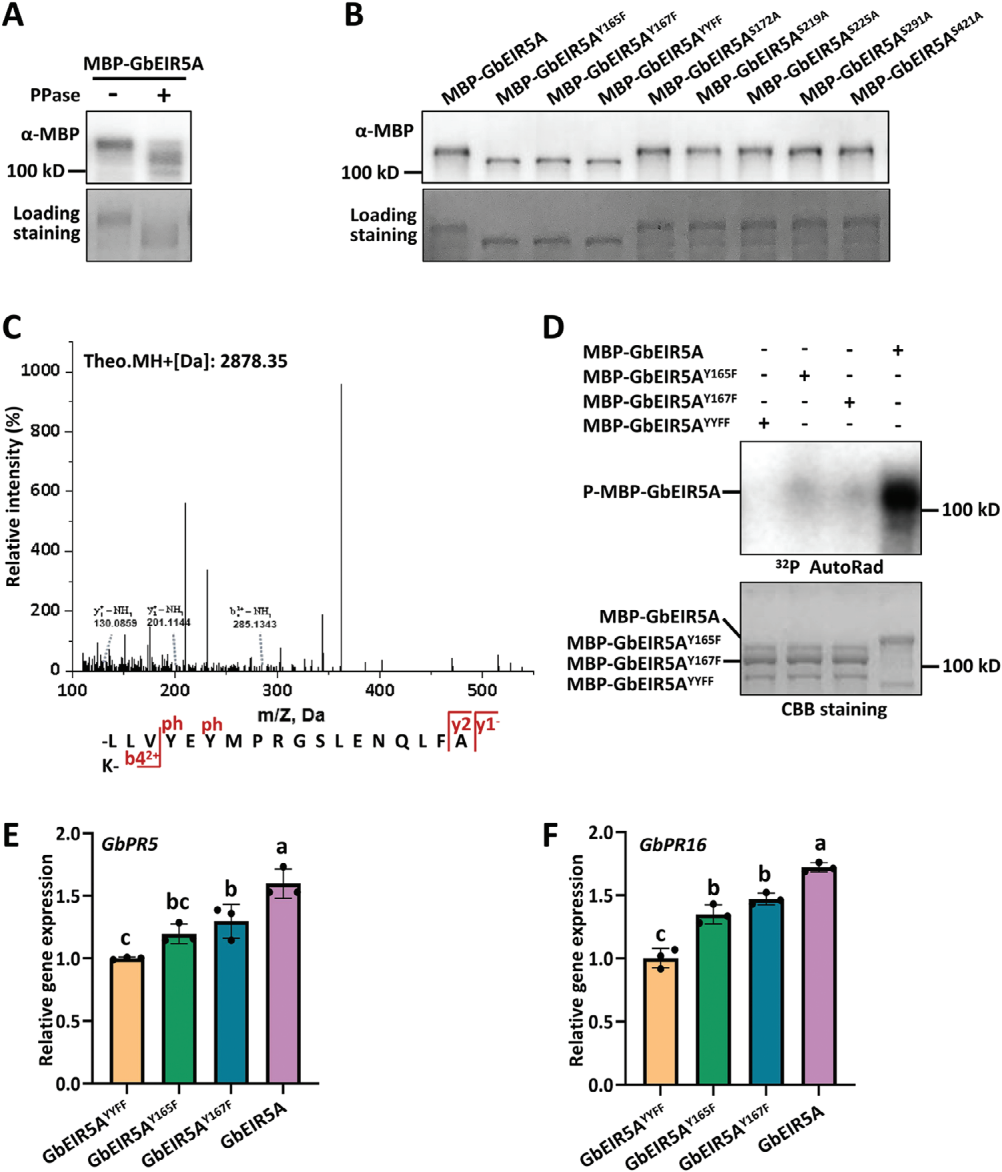
GbEIR5A phosphorylation regulates the expression of resistance‐related genes. A) PPase treatment dephosphorylated GbEIR5A. Recombinant proteins were treated with or without 2 units/mL PPase as indicated. B) Tyr165 and Tyr167 are required for GbEIR5A full phosphorylation. MBP‐fused recombinant proteins were expressed in *Escherichia coli* BL21 strain. Protein samples were separated by SDS‐PAGE and subjected to anti‐MBP immunoblotting. C) PAMP induces GbEIR5A phosphorylation at Tyr165 and Tyr167 residues in vivo. Mass spectrometry analysis identified the phosphorylation of GbEIR5A at Tyr165 and Tyr167 residues. GbEIR5A‐HA was transiently expressed in *Arabidopsis* protoplasts. Total protein was extracted from protoplasts treated with flg22. GbEIR5A‐HA was immunoprecipitated by anti‐HA and subjected to mass spectrometry analysis. D) GbEIR5A phosphorylates in vitro. Recombinant proteins of MBP‐GbEIR5A, MBP‐GbEIR5A^Y165F^, MBP‐GbEIR5A^Y167F^, and MBP‐GbEIR5A^YYFF^ were purified and subjected to in vitro phosphorylation assays. CBB staining indicates the loading of the protein. E,F) Transient expression of GbEIR5A^Y165F^, GbEIR5A^Y167F^, or GbEIR5A^YYFF^ reduces the expression of resistance‐related genes in cotton. Cotton leaves were infiltrated with *Agrobacterium* carrying the indicated constructs. Total RNA from infiltrated plants was extracted for RT‐qPCR analyses of *GbPR5* E) and *GbPR16* F) expression at 48 hours post‐inoculation. Values are the means ± SD; *n* = 3. Error bars indicate the standard deviation of 3 biological replicates. The experiments were repeated 3 times with similar results. Different letters indicate significant differences, as determined by one‐way ANOVA.

Phosphorylation plays a crucial role in RLCKs in regulating signal transduction and cellular activities. The mobility of GbEIR5A was reduced by lambda protein phosphatase (PPase) treatment (**Figure** **6**A), suggesting that GbEIR5A undergoes phosphorylation modification. *GbEIR5A* was then cloned into *pUC‐35S‐GbEIR5A‐HA*, expressed in *Arabidopsis* protoplasts, and treated with PAMP. Phosphorylated GbEIR5A was fractionated by SDS‐PAGE and subjected to mass spectrometry analyses to characterize putative in planta phosphorylation sites. Tyr165, Tyr167, Ser172, Ser219, Ser225, Ser291, and Ser421 were identified as potential residues that are phosphorylated by PAMP in planta. The residues were then mutated and subjected to a mobility shift assay. A compromised phosphorylation was observed when Tyr165 and Tyr167 were mutated to phenylalanine alone (GbEIR5A^Y165F^ and GbEIR5A^Y167F^) or Tyr165 and Tyr167 were mutated to phenylalanine simultaneously (GbEIR5A^YYFF^; Figure 6B,C). The recombinant proteins of GbEIR5A, GbEIR5A^Y165F^, GbEIR5A^Y167F^, and GbEIR5A^YYFF^ were purified for in vitro phosphorylation assay. The results showed that both GbEIR5A^Y165F^ and GbEIR5A^Y167F^ exhibited reduced phosphorylation activity compared to GbEIR5A, while GbEIR5A^YYFF^ significantly compromised the phosphorylation of GbEIR5A (Figure 6D). The results indicate that Tyr165 and Tyr167 contribute to GbEIR5A phosphorylation. The upregulation of *pathogenesis‐related* (*PR*) genes serves as a defense strategy employed by plants to address both biotic and abiotic stress factors. We next expressed GbEIR5A, GbEIR5A^Y165F^, GbEIR5A^Y167F^, and GbEIR5A^YYFF^ in cotton cotyledons, and examined the expression of *GbPR5* and *GbPR16*. The results showed that the expression of *GbPR5* and *GbPR16* was significantly reduced when expressing GbEIR5A^Y165F^, GbEIR5A^Y167F^, or GbEIR5A^YYFF^, compared to expressing GbEIR5A (Figure 6E,F). These observations thus indicate that Tyr165 and Tyr167 are critical residues required for resistance in cotton.

### 2.6 VdEPD1 Enhances the Expression of *GbEIR5A/D* to Potentiate PTI

**Figure 7:**
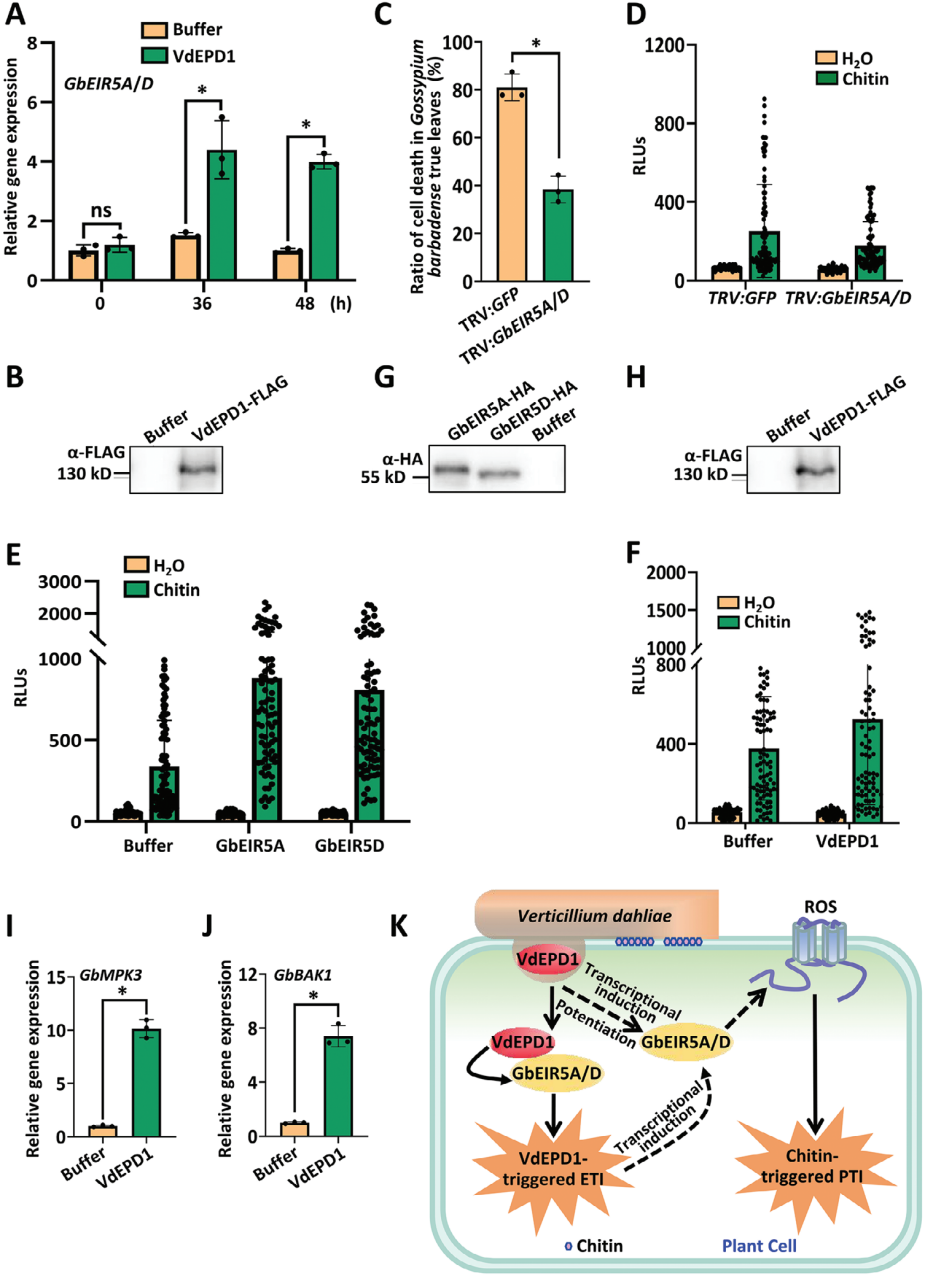
VdEPD1 potentiates GbEIR5A/D to enhance cotton PTI. A) The expression levels of *GbEIR5A*/*D* are induced in *G*. *barbadense* plants infiltrated with *Agrobacterium* carrying *pCambia1300‐35S‐VdEPD1‐FLAG* (OD_600_: 0.8). Total RNA from infiltrated plants was extracted for RT‐qPCR analysis of *GbEIR5A/D* expression at 36 or 48 hours post‐inoculation. *GbUBQ7* was used as an internal control. Values are the means ± SD; *n* = 3. Error bars indicate the standard deviation of three biological replicates. B) Expression level of VdEPD1‐FLAG protein in *G*. *barbadense*. *G*. *barbadense* leaves were infiltrated with the *Agrobacterium* strain as indicated. Total protein was extracted for anti‐FLAG western blotting. C) Silencing of *GbEIR5A*/*D* compromises VdEPD1‐induced cell death. *Agrobacterium* carrying *pCambia1300‐35S‐VdEPD1‐FLAG* (OD_600_: 0.8) was infiltrated into *GFP*‐silenced or *GbEIR5A/D*‐silenced *G*. *barbadense* plants. The percentage of cell death was recorded at 5 days post‐inoculation. Values are the means ± SD; *n* = 3. Error bars indicate the standard deviation of three biological replicates. Student's *t*‐test was carried out to determine the significance of the difference. * indicates a significant difference at a *p*‐value of <0.05. ns indicates no significant difference. D) *GbEIR5A/D*‐silienced plants are compromised in PAMP‐induced oxidative burst in *G*. *barbadense* plants. E) GbEIR5A and GbEIR5D enhanced PAMP‐induced oxidative burst in *G*. *barbadense* plants. F) VdEPD1 enhanced PAMP‐induced oxidative burst in *G*. *barbadense* plants. Leaf strips from silenced or overexpressed plants were incubated in H_2_O overnight. 500 µg/mL chitin was added, and the ROS production was immediately measured using a luminol‐based assay. Relative amounts of H_2_O_2_ are shown as relative luminescence units. G,H) Expression levels of GbEIR5A‐HA, GbEIR5D‐HA G), and VdEPD1‐FLAG H) proteins in *G*. *barbadense* plants used for ROS detection. *G*. *barbadense* leaves were infiltrated with the *Agrobacterium* strain as indicated. Total protein was extracted for anti‐FLAG or anti‐HA western blotting. I,J) Transient expression of VdEPD1 induced the expression of PTI‐related genes in *G*. *barbadense*. *G*. *barbadense* leaves were infiltrated with *Agrobacterium* carrying *pCambia1300‐35S‐VdEPD1‐FLAG* (OD_600_: 0.8). Total RNA from infiltrated plants was extracted for RT‐qPCR analyses of *GbMPK3* I) and *GbBAK1* J) expression at 3 days post‐inoculation. Values are the means ± SD; *n* = 3. Error bars indicate the standard deviation of 3 biological replicates. The experiments were repeated three times with similar results. Student's *t*‐test was carried out to determine the significance of the difference. ^*^ indicates a significant difference at a *p*‐value of <0.05. K) Recognition of a fungal effector potentiates PAMP‐triggered immunity in cotton. Expression of VdEPD1 leads to the accumulation of ROS and the activation of cell death in cotton plants. VdEPD1 interacts with GbEIR5A and GbEIR5D, inducing their expression levels. Silencing *GbEIR5A*/*D* significantly impairs VdEPD1‐triggered cell death in cotton plants. *GbEIR5A/D*‐silenced cotton plants exhibit compromised ROS accumulation triggered by chitin, whereas overexpression of *GbEIR5A* or *GbEIR5D* enhances chitin‐induced ROS production.

The expression of *NbEIR* is induced by VdEPD1 (Figure 4E). To determine whether the expression of *GbEIR5A/D* is also induced by VdEPD1, the cotton cotyledons were inoculated with *Agrobacterium* carrying *pCambia1300‐35S‐VdEPD1‐FLAG*. The results demonstrate that the expression of *GbEIR5A/D* is significantly induced by VdEPD1, indicating its ability to up‐regulate the transcriptional levels of these two genes (**Figure** **7**A). The expression of VdEPD1 protein was confirmed by immunoblotting (Figure 7B). To further determine whether VdEPD1‐induced cell death in *G. barbadense* requires GbEIR5A/D, we infiltrated *Agrobacterium* carrying *pCambia1300‐35S‐VdEPD1‐FLAG* into the leaves of *GbEIR5A/D*‐silenced cotton plants. As expected, silencing of *GbEIR5A/D* impaired VdEPD1‐induced cell death (Figure 7C), suggesting that GbEIR5A and GbEIR5D are involved in VdEPD1‐induced ETI. Taken together, the above results indicate that *GbEIR5A/D* are significantly up‐regulated by VdEPD1 and involved in VdEPD1‐induced ETI.

To explore whether GbEIR5A/D contributes to cotton PTI, we examined ROS burst in *GbEIR5A/D*‐silenced cotton plants in response to chitin. The results indicated that silencing of *GbEIR5A/D* significantly compromised chitin‐induced ROS production (Figure 7D). In support of a positive contribution of GbEIR5A/D to PTI, overexpressing GbEIR5A and GbEIR5D further enhances chitin‐induced ROS production (Figure 7E). These results indicate that GbEIR5A and GbEIR5D contribute positively to PTI. Furthermore, overexpression VdEPD1 in cotton significantly enhanced the chitin‐induced ROS burst (Figure 7F). The expression of GbEIR5A, GbEIR5D, and VdEPD1 proteins in *G. barbadense* plants used for ROS detection was confirmed by immunoblotting (Figure 7G,H). Overexpression of VdEPD1 in cotton significantly enhanced the expression of PTI‐responsive genes such as *GbMPK3* ^[^ ^57^ ^]^ and G*bBAK1* ^[^ ^58^ ^]^ (Figure 7I,J). The above results indicate that VdEPD1 potentiates GbEIR5A/D to enhance cotton PTI. In addition, the transcriptional activation of GbEIR5A/D could also be downstream of VdEPD1‐induced cell death (Figure 7K). The protein sequences of GbEIR5A/D exhibit high similarity to *Arabidopsis* RIPK (Figure S15A, Supporting Information). *P. syringae* effector AvrB targets RIPK, causing the phosphorylation of RIN4, which subsequently triggers the activation of RPM1 and elicits ETI.^[^ ^38^, ^39^, ^40^ ^]^ We measured the production of ROS in Col‐0 and the *ripk* mutant upon treatment with flg22. The results showed that ROS production was reduced in the *ripk* mutant compared with Col‐0 (Figure S15B, Supporting Information), indicating that RIPK regulates the ROS burst triggered by PAMPs.^[^ ^39^ ^]^ Therefore, we speculate that RIPK may respond to VdEPD1‐triggered defenses. To test this speculation, the Col‐0 and the *ripk* mutant were inoculated with both V592 and the Vd∆*epd1* mutant. When compared with V592, the Vd∆*epd1* mutant displayed enhanced virulence on the Col‐0 plants. However, this enhanced virulence resulting from the deletion of VdEPD1 was partially impaired in the *ripk* mutant plants compared to the Col‐0 plants (Figure S15C,D, Supporting Information). The results indicate that RIPK contributes to the recognition of VdEPD1 in *Arabidopsis*.

## 3 Discussion

Cotton is a crucial economic crop, yet its production is threatened by various diseases. Among these, Verticillium wilt, caused by the fungus *V. dahliae*, poses a significant challenge to cotton cultivation due to its difficulty in prevention.^[^ ^59^ ^]^ In plants, PTI and ETI are the two major immune mechanisms governing defense against pathogens. Complex interplays between PTI and ETI in the model plant *Arabidopsis thaliana* have been demonstrated.^[^ ^3^, ^4^, ^5^, ^6^ ^]^ Currently, there is a considerable gap in our understanding of the molecular recognition mechanisms between cotton and *V. dahliae*. Furthermore, the intricate framework of cotton's immune signaling pathway remains elusive, requiring further investigation. Our research discovered that an effector, VdEPD1, encoded by *V. dahliae*, possesses elicitor activity (Figure 3A,B; Figure S3A, Supporting Information); cotton GbEIR5A/D is able to recognize VdEPD1 and activate ETI (Figures 5A,B,F,G and 7C); VdEPD1 induces the expression of *GbEIR5A/D* at the transcriptional level (Figure 7A), and GbEIR5A/D positively regulates chitin‐activated PTI (Figure 7E,F). This research has elucidated the function of GbEIRs in cotton immune regulation and indicated GbEIR‐mediated potentiation of PTI by ETI in cotton.

Pathogens manipulate plant immunity by secreting effectors to promote pathogenicity. Some of these effectors are recognized by plants and trigger ETI, while most effectors possess secretory signals. However, there are also effectors that lack secretory peptides but are secreted and transported through atypical pathways, such as VdIsc1 from *V. dahliae*.^[^ ^60^ ^]^ Although VdEPD1 lacks a typical signal peptide, we found that VdEPD1 localizes at the hyphal neck and forms ring signals in *V. dahliae*. The self‐assembling split NanoLuc‐based assay and subcellular localization analyses indicate that VdEPD1 is secreted through an atypical pathway, and this process depends on the exocyst complex (Figure 3M,O).

Previous studies have demonstrated that RLCKs are key regulators of PTI in *Arabidopsis*.^[^ ^61^ ^]^ In addition, some RLCKs play an important role in the recognition of effectors, and the targeting and modification of these effectors by RLCKs can be monitored by NLRs, thereby activating ETI.^[^ ^39^, ^40^, ^46^, ^47^, ^48^, ^49^, ^50^, ^51^, ^52^, ^62^, ^63^ ^]^ In the model plant *Arabidopsis*, PTI and ETI potentiate each other;^[^ ^3^, ^4^, ^5^, ^6^ ^]^ However, the framework of PTI and ETI signaling pathways in cotton has not yet been established. Our research suggests a model in which the recognition of the effector VdEPD1 by cotton enhances the transcription level of *GbEIR5A/D*, thereby promoting GbEIR5A/D‐mediated PTI, providing molecular evidence for the potentiation of PTI by ETI in cotton. In addition, unlike the modification of RLCKs by effectors in existing studies, the induction of *GbEIR5A/D* transcription by effectors suggests a new mode of potentiation between PTI and ETI, in addition to protein–protein interactions. How VdEPD1 induces the upregulation of *GbEIR5A/D* transcription level is still unclear. This is one of the issues that needs to be studied in the future. The protein with the highest similarity to cotton GbEIR5A/D in *Arabidopsis* is RIPK, which plays a role in both *P. syringae* effector AvrB‐mediated ETI and flg22‐induced PTI, indicating that RIPK is a cross‐talk node between ETI and PTI in *Arabidopsis*.

## 4 Experimental Section

### Plant Materials and Constructs

The virulent *V. dahliae* strain V592^[^ ^26^, ^42^, ^64^, ^65^ ^]^ isolated from cotton in Xinjiang, China, was used in this study. Other strains used were constructed from V592. The T‐DNA insertional mutant library was constructed previously.^[^ ^42^ ^]^ All the strains were first grown on potato dextrose agar (PDA) medium for a week at 25 °C in the dark. The conidia for infection assays were collected from potato dextrose broth (PDB) medium with shaking at 150 rpm at 25 °C for 3 days. Fungal transformation, DNA extraction, and Southern blot were performed as previously described.^[^ ^13^ ^]^ The Verticillium wilt‐resistant island cotton Hai7124 (*G. barbadense*)^[^ ^53^ ^]^ and *Arabidopsis thaliana* Col‐0 wild‐type plants were used in this study. Constructs used in this study include knockout constructs *pKOVdEPD1* and *pKOVd01961*, complementary construct *pNEO‐VdEPD1com*, plant expression constructs *pCambia1300‐35S‐VdEPD1‐FLAG*, *pCambia1300‐35S‐RPS2‐FLAG*, *pCambia1300‐35S‐VdEPD1‐NLuc‐HA*, *pCambia1300‐35S‐NbEIR‐CLuc*, *pCambia1300‐35S‐GbEIR5A‐CLuc*, *pCambia1300‐35S‐GbEIR5D‐CLuc*, *pCambia1300‐35S‐GbEIR5A‐mCherry*, *pCambia1300‐35S‐VdEPD1‐GFP*, *pUC‐35S‐GbEIR5A‐HA*, *pUC‐35S‐GbEIR5A‐FLAG*, *pUC‐35S‐GbEIR5D‐FLAG*, *pUC‐35S‐VdEPD1‐HA*, *pUC‐35S‐GbEIR5A‐mCherry*, *pUC‐35S‐VdEPD1‐GFP*, etc., and virus‐induced gene silencing constructs *pTRV2‐NbEIR*, *pTRV2‐GbEIR5A/D*, etc. Primer sequences are provided in Table S1 (Supporting Information).

### Infection Assays

Cotton plants were infected by the root‐dip inoculation method.^[^ ^42^ ^]^ A conidial suspension of 10^6^/mL from the indicated strain was used as the inoculum. The disease grade was classified as follows: 0 (no observed symptoms), 1 (0–25% wilted leaves), 2 (25–50%), 3 (50–75%), and 4 (75–100%).^[^ ^20^ ^]^ The onion epidermis infection assay was performed as described.^[^ ^66^ ^]^ A conidial suspension of 10^7^/mL from the V592/*GFP* and V592/*VdEPD1‐GFP* strains was inoculated onto the inner layer of onion epidermal cells and incubated on 1% water agar plates for 3–5 days before confocal imaging.

### Transient Expression in N. benthamiana or Cotton Plants

*Agrobacterium tumefaciens* strain GV3101 carrying the indicated constructs or “Buffer” [10 mm MgCl_2_, 10 mm MES (pH 5.7), and 200 µm acetosyringone] was infiltrated into the leaves of *N. benthamiana* or cotton plants. The leaves were collected for gene expression or immunoblotting analyses.

### RNA Extraction and RT‐qPCR

Total RNA was extracted from *V. dahliae* using the TRIzol reagent (Invitrogen) and from *N. benthamiana* or cotton plants using the AFTMag quick complex plant RNA extraction kit (ABclonal). cDNA was reverse transcribed using HiScript II Q RT Supermix (Vazyme Biotech Co., Ltd) and RT‐qPCR was performed using ChamQ SYBR qPCR Master Mix (Vazyme Biotech Co., Ltd) by the Bio‐Rad CFX96 Real‐Time system. *NbACTIN*, *GbUBQ7*, and *VdELF1* genes were used as internal controls for the RT‐qPCR analysis in *N. benthamiana*,^[^ ^67^ ^]^ *G*. *barbadense*,^[^ ^57^ ^]^ and *V. dahliae*,^[^ ^64^ ^]^ respectively, following the literature. The gene‐specific primers are listed in Table S1 (Supporting Information).

### Virus‐induced Gene Silencing in N. benthamiana or Cotton Plants

*pTRV1*, *pTRV2‐GFP*, *pTRV2‐NbEIR*, *pTRV2‐GbEIR5A/D*, and other constructs were transformed into *Agrobacterium* by electroporation. The *Agrobacterium* strain carrying *pTRV1*, together with the *Agrobacteriu* *m* strains carrying *pTRV2‐GFP*, *pTRV2‐NbEIR*, *pTRV2‐GbEIR5A/D*, etc., were infiltrated into the four‐leaf stage *N. benthamiana* or cotyledons of the cotton plants.

### Split‐Luciferase Complementation Assay

*Agrobacterium* strains carrying constructs that are tagged with either NLuc or CLuc were infiltrated into the leaves of four‐week‐old *N. benthamiana* plants. After 2 days, the LUC activity within the leaves was assayed.

### Protein Extraction

*N. benthamiana* or cotton leaves were harvested at 36–48 hours post‐inoculation with *Agrobacterium*. Total protein was extracted using extraction buffer containing 50 mm HEPES (pH 7.5), 150 mm KCl, 1 mm EDTA, 1 mm DTT, 1% Triton X‐100, and 1 × proteinase inhibitor cocktail.

### Co‐Immunoprecipitation Assay in Protoplasts

Five‐week‐old *Arabidopsis* plants were used for protoplast isolation. *pUC‐35S‐VdEPD1‐HA* was transfected alone or co‐transfected with *pUC‐35S‐GbEIR5A‐FLAG* or *pUC‐35S‐GbEIR5D‐FLAG*, into *Arabidopsis* protoplasts. Total protein was extracted using an extraction buffer. For anti‐FLAG IP, total protein was incubated with FLAG beads at 4 °C for 4 hours. The agarose beads were collected and boiled for 5 min with 1× protein loading buffer. Immunoprecipitates were separated by 8% SDS‐PAGE, and the presence of VdEPD1‐HA, GbEIR5A‐FLAG, or GbEIR5D‐FLAG was detected by anti‐HA or anti‐FLAG immunoblotting.

### Fluorescence Microscopy

The *Agrobacterium* strain carrying *pCambia1300‐35S‐GbEIR5A‐mCherry* was infiltrated alone or together with the *Agrobacterium* strain carrying *pCambia1300‐35S‐VdEPD1‐GFP* into the leaves of four‐week‐old *N. benthamiana*. GFP and mCherry fluorescence were observed using a Leica SP8 confocal laser scanning microscope system 2 days postinfiltration. For fluorescence microscopy in *G*. *barbadense* protoplasts, the protoplasts were co‐transfected with *pUC‐35S‐VdEPD1‐GFP* and *pUC‐35S‐GbEIR5A‐mCherry* and incubated overnight under faint light before observing the GFP and mCherry fluorescence. To observe the localization of the VdEPD1‐GFP protein, the mycelia were grown on MM medium overlaid with cellophane for 3 days, and the plasma membrane was stained with FM4‐64 (Thermo Fisher) according to the manufacturer's protocol. Fluorescent photographs were taken using a Leica SP8 confocal laser scanning microscope system. The pieces of cellophane with mycelium were collected and observed as described.^[^ ^14^ ^]^

### Transcriptome Analysis

Leaves of *N. benthamiana* were infiltrated with buffer or *Agrobacterium* strain carrying *pCambia1300‐35S‐VdEPD1‐FLAG* suspension for 34 hours. Total RNA was extracted as described above. Transcriptome data analysis was performed by Berry Genomics Corporation, Beijing, China.

### Self‐Assembling Split NanoLuc‐Based Assay

*pNEO‐VdEPD1‐HiBiT* was introduced into *V. dahliae* V592 and the VdΔ*exo70* mutant. The supernatant obtained from the culture media of the V592, V592/*VdEPD1‐HiBiT*, and VdΔ*exo70*/*VdEPD1‐HiBiT* strains was incubated with the recombinant protein NanoLucN‐His, along with 10 µm of coelenterazine in a 96‐well plate. Subsequently, the luminescence was monitored using an EnSpire Multimode Plate Reader.

### ROS Measurement

ROS accumulation in *N. benthamiana* was visualized by diaminobenzidine (DAB) staining. The treated *N. benthamiana* leaves were incubated in DAB solution for 2–12 hours and destained with 95% ethanol. Additionally, cotton leaves were sliced into ≈2 mm strips, and incubated in H_2_O for 12 hours, and equal amounts of leaf tissue were treated with 500 µg/mL chitin solution containing luminol and horseradish peroxidase (Sigma). Luminescence was recorded for 20 min.

### Assays for Botrytis cinerea and Phytophthora Capsici infection

Four‐week‐old *N. benthamiana* leaves were infiltrated with *Agrobacterium* harboring VdEPD1 or buffer, as indicated. The leaves were inoculated with Botrytis cinerea or Phytophthora capsici 1 day post‐inoculation with the *Agrobacterium* strain carrying *pCambia1300‐35S‐VdEPD1‐FLAG*, as indicated. *B. cinerea* strain B05.10 was grown on Malt Extract Agar (Oxoid, 50 g/L). Conidia were harvested, and two droplets of conidial suspension (5 µL, 10^6^ conidia/mL in PDB) were inoculated on the infiltrated leaves.^[^ ^68^ ^]^ The *P. capsici* mycelial plug with a diameter of 5 mm was inoculated on the infiltrated leaves. The inoculated plants were placed in an incubator at 25 °C and 80% relative humidity.

### Mass Spectrometric Analysis

GbEIR5A‐HA was transfected into *Arabidopsis* protoplasts isolated from five‐week‐old plants, and treated with flg22 for 5 min. Total protein was extracted from protoplasts and immunoprecipitated with anti‐HA. Immunoprecipitates were separated by SDS‐PAGE and silver‐stained. Mass spectrometric data analysis was performed by Beijing Qinglian Biotech Co., Ltd. Database searches were performed on an in‐house Mascot server (Matrix Science Ltd., London, UK) against the GbEIR5A protein sequence. All identified phosphorylated peptides were manually checked to exclude false positives.

### Statistical Analysis

The preprocessing of data and sample size (n) for each statistical analysis are listed in the figure legends. Error bars in the figures represent the standard deviation (SD) of the mean. Statistical analysis was performed using the GraphPad Prism software. The two‐tailed paired Student's *t*‐test was used to determine the significant difference between the two groups, and a one‐way ANOVA test was used to determine the significant difference among more than two groups. Asterisks (*) or letters (a/b/c) indicate statistical significance at *p* < 0.05.

## Conflict of Interest

The authors declare no conflict of interest.

## Supporting information



Supporting Information

Supplemental Table 1

## Data Availability

Research data are not shared.
