# Supplementary material for: Recognition of a Fungal Effector Potentiates Pathogen‐Associated Molecular Pattern‐Triggered Immunity in Cotton
Source: Adv Sci (Weinh). 2024 Nov 3;12(1):2407787. doi: 10.1002/advs.202407787 (PMC11714242; doi:10.1002/advs.202407787)
Supplement: Supplementary file 1 — Supporting Information [file ADVS-12-2407787-s002.pdf]

ADVANCED SCIENCE
Open Access

Supporting Information

for *Adv. Sci.*, DOI 10.1002/advs.202407787

Recognition of a Fungal Effector Potentiates Pathogen-Associated Molecular Pattern-Triggered Immunity in Cotton

*Lifan Sun, Xiangguo Li, Jiajie Zhong, Yu Wang, Baiyang Li, Ziqin Ye and Jie Zhang**

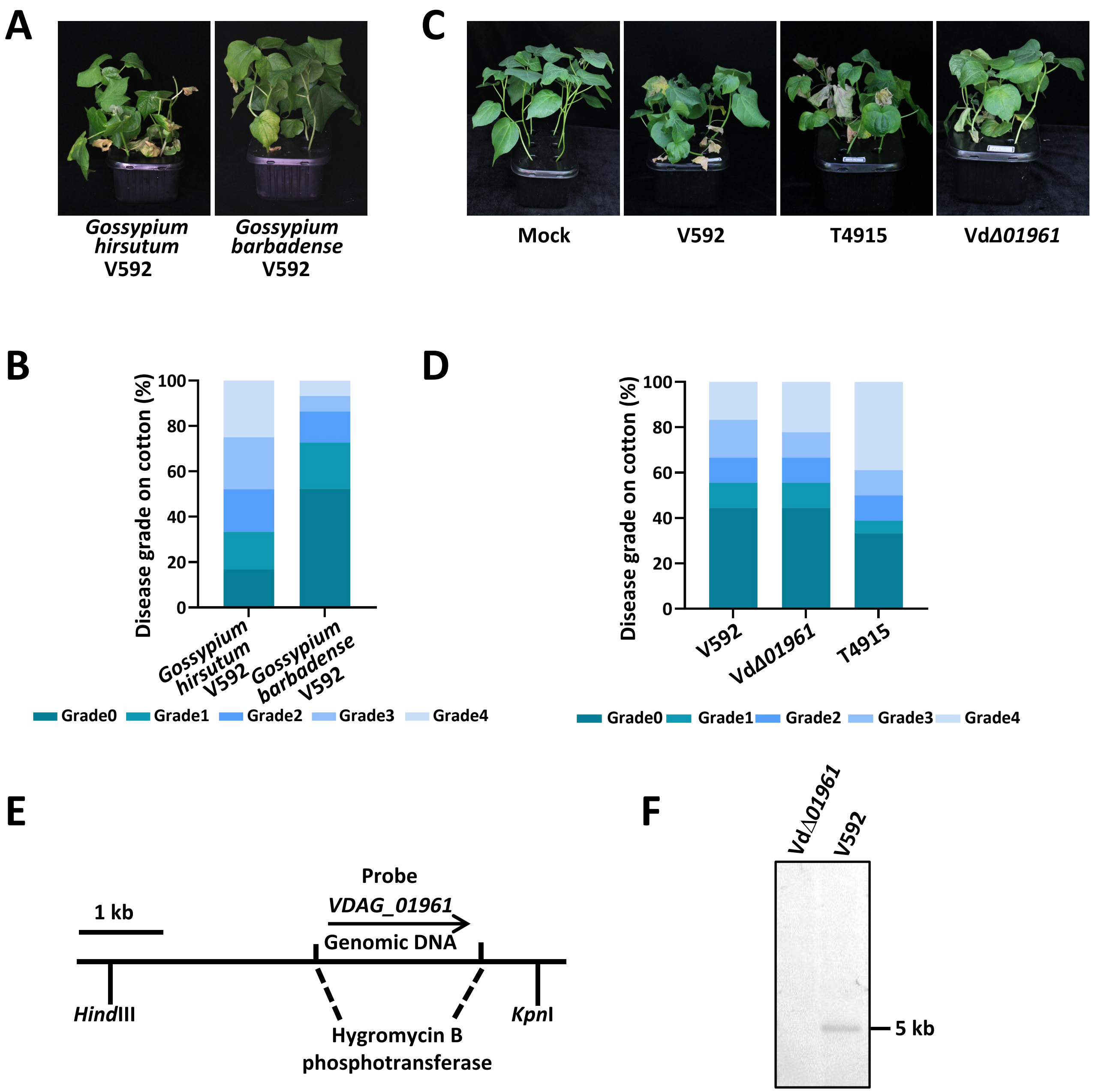

Figure S1. VdΔ*01961* mutant exhibits comparable pathogenicity as that of WT strain.

A and **B**, *Gossypium barbadense* exhibits greater resistance to *Verticillium dahliae* compared to *Gossypium hirsutum*. Disease symptoms (**A**) and index analyses (**B**) of *G. barbadense* and *G. hirsutum* infected with V592 strain. **C** and **D**, Disease symptoms (**C**) and index analyses (**D**) of cotton infected with V592, VdΔ*01961*, or T4915 strains. Three-week-old cotton seedlings were inoculated with V592, VdΔ*01961*, or T4915. The plants were photographed and subjected to disease index analyses 4-5 weeks post-inoculation. Disease indexes were evaluated with 3 replicates generated from 18 plants (*n* = 18) for each inoculum. **E**, Schematic description of *VDAG_01961* gene deletion. **F**, Southern blot analysis of the *VDAG_01961* gene deletion in the VdΔ*01961* mutant. Genomic DNA samples isolated from V592 and VdΔ*01961* strains were digested with *Hind*III and *Kpn*I and subjected to Southern blot analysis.

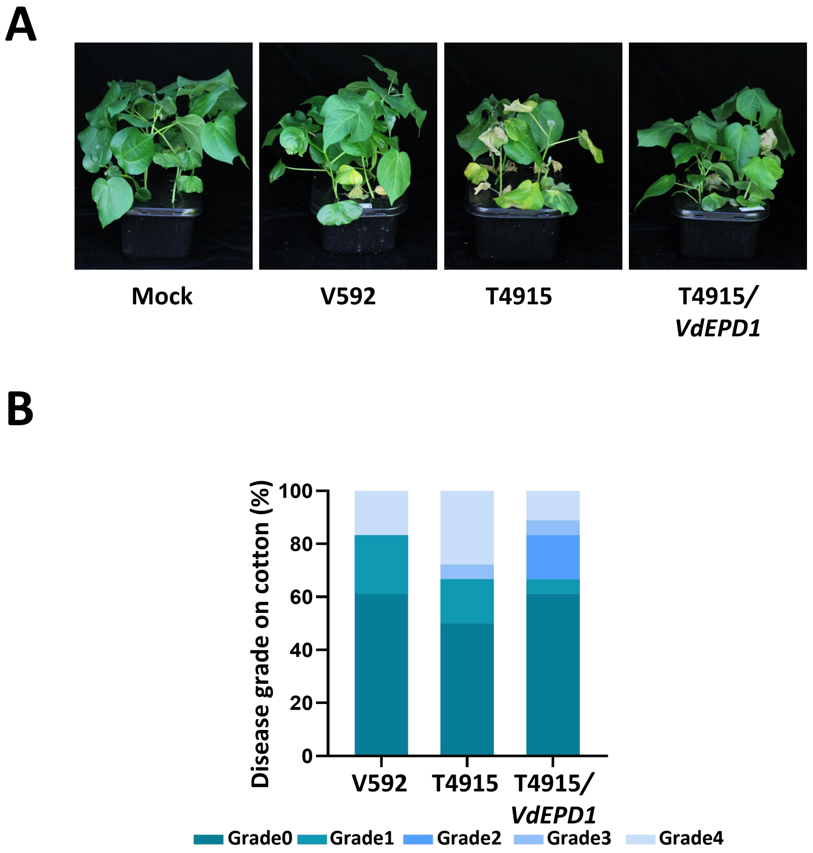

Figure S2. *VdEPD1* suppresses the enhanced pathogenicity of the T4915 mutant.
A and **B**, Disease symptoms (**A**) and index analyses (**B**) of cotton infected with V592, T4915, or T4915/*VdEPD1* strains. Three-week-old cotton seedlings were inoculated with V592, T4915, or T4915/*VdEPD1*. The plants were photographed and subjected to disease index analyses 4-5 weeks post-inoculation. Disease indexes were evaluated with 3 replicates generated from 18 plants (n = 18) for each inoculum.

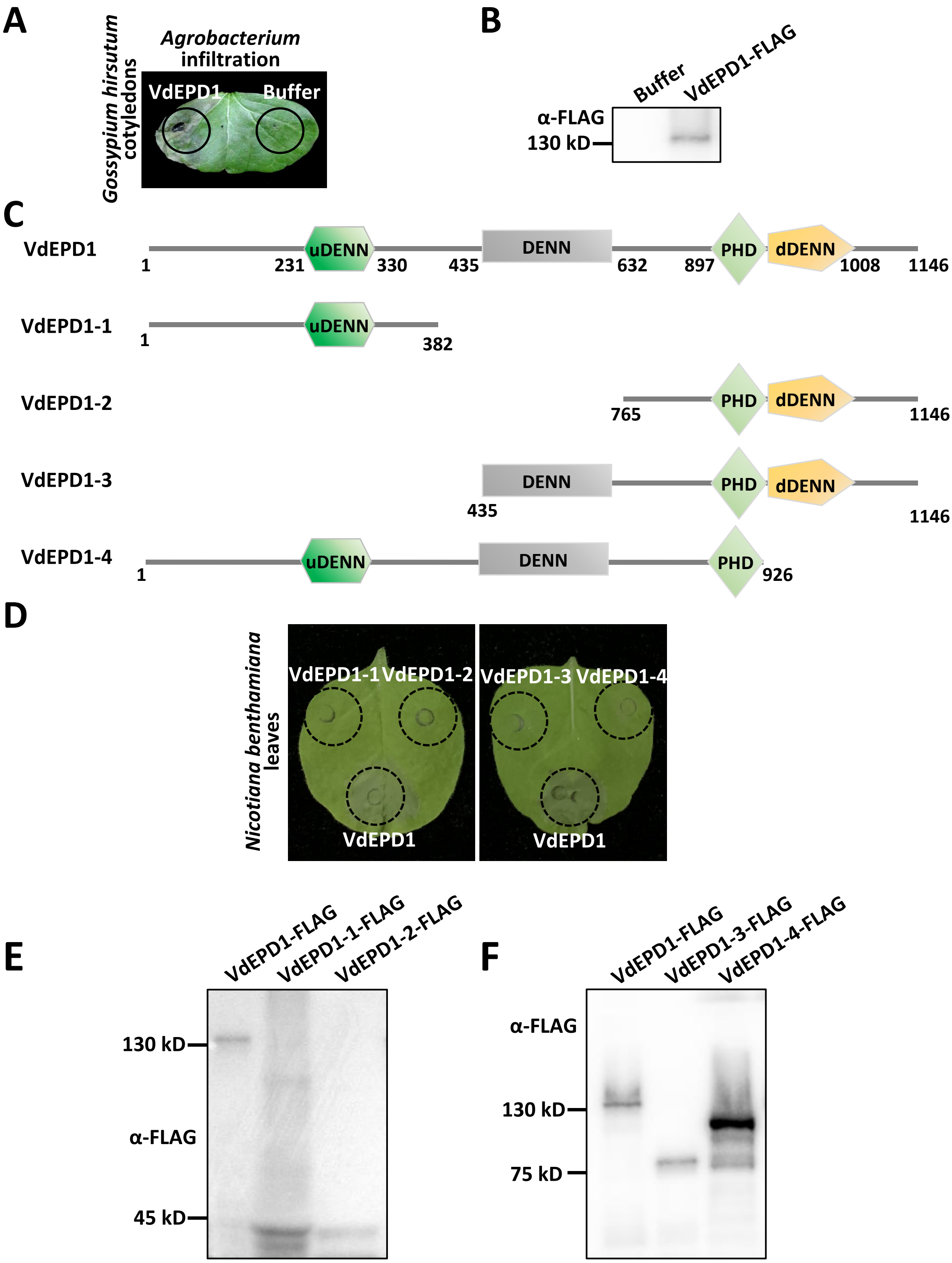

Figure S3. The full-length protein of VdEPD1 is required for inducing cell death.
A, Transient expression of VdEPD1 induced cell death in *G. hirsutum*. *Agrobacterium* carrying the *pCambia1300-35S-VdEPD1-FLAG* (OD_{600}: 0.8) was infiltrated into *G. hirsutum* leaves. **B**, Expression of VdEPD1 in *G. hirsutum. G. hirsutum* leaves were infiltrated with *Agrobacterium* strain as indicated. Total protein was extracted for anti-FLAG western blotting. **C**, Schematic diagram of VdEPD1 and its truncated variants. **D**, uDENN, DENN, dDENN, and PHD are required for VdEPD1-induced cell death in plants. *N. benthamiana* leaves were infiltrated with *Agrobacterium* carrying the indicated construct. The photograph was taken at 2 days post-inoculation. **E** and **F**, Expression of VdEPD1-FLAG, VdEPD1-1-FLAG, VdEPD1-2-FLAG, VdEPD1-3-FLAG, and VdEPD1-4-FLAG in *N. benthamiana*. *N. benthamiana* leaves were infiltrated with *Agrobacterium* strain as indicated. Total protein was extracted for anti-FLAG western blotting.

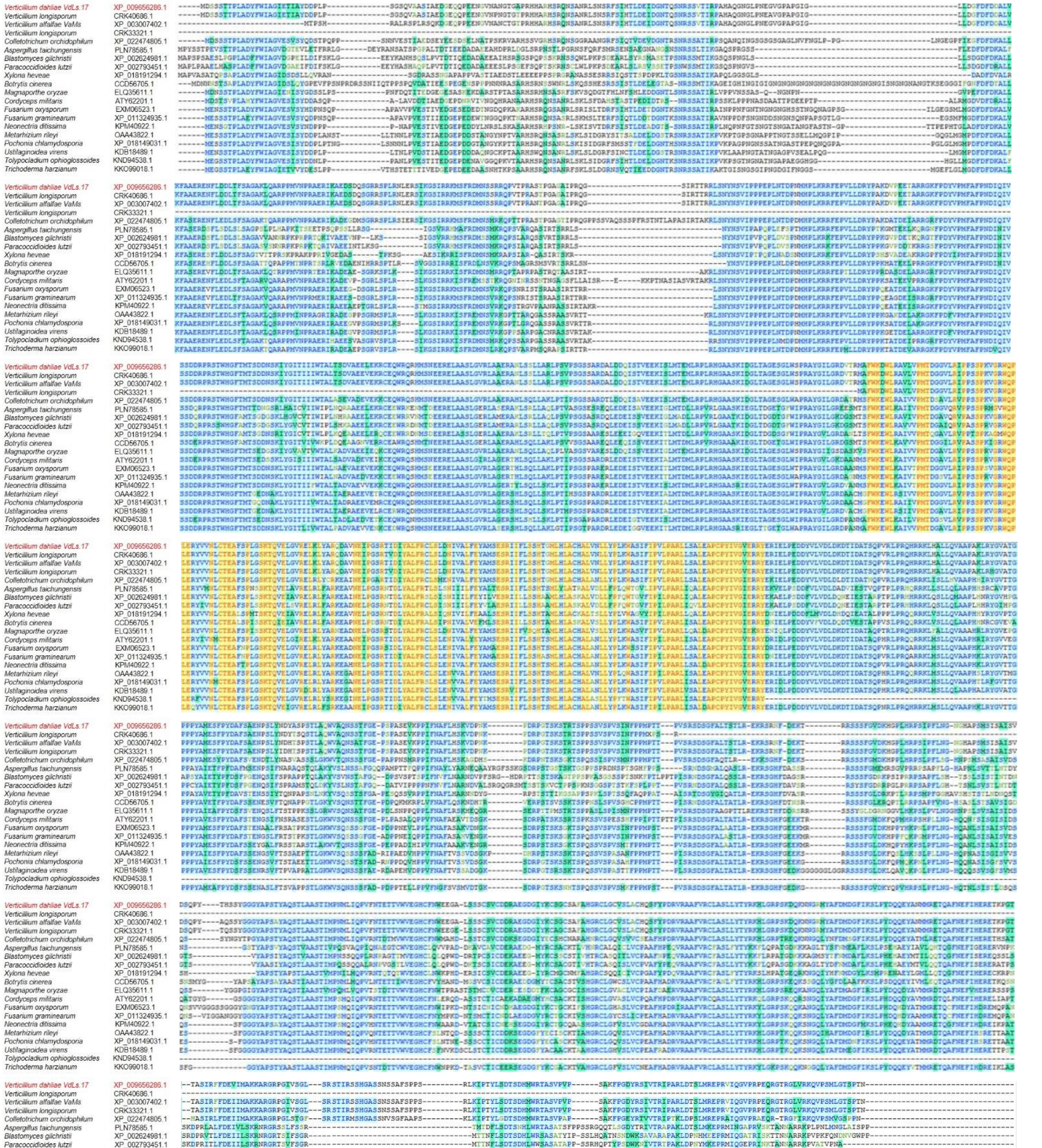

Figure S4. An alignment of VdEPD1 homologous proteins.

VdEPD1 homologous proteins in *Verticillium dahliae* VdLs.17, *Verticillium longisporum*, *Verticillium alfalfae* VaMs, *Colletotrichum orchidophilum*, *Aspergillus taichungensis*, *Blastomyces gilchristii*, *Paracoccidioides lutzii*, *Xylona heveae*, *Botrytis cinerea*, *Magnaporthe oryzae*, *Cordyceps militaris*, *Fusarium oxysporum*, *Fusarium graminearum*, *Neonectria ditissima*, *Metarhizium rileyi*, *Pochonia chlamydosporia*, *Ustilaginoidea virens*, *Tolypocladium ophioglossoides* and *Trichoderma harzianum* were aligned using Vector NTI software.

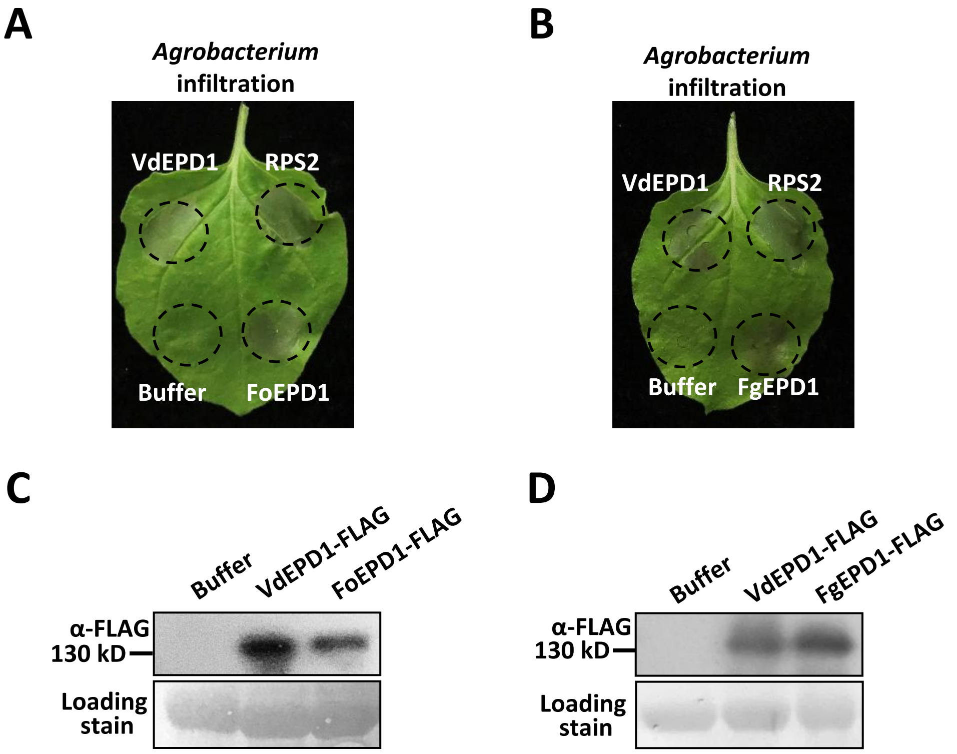

Figure S5. Homologues of VdEPD1, *Fusarium oxysporum* (FoEPD1) and *Fusarium graminearum* (FgEPD1), can trigger defense in *N. benthamiana*.

A and **B**, Transient expression of FoEPD1 and FgEPD1 induced cell death in *N. benthamiana*. *Agrobacterium* strains carrying the *FoEPD1*, *FgEPD1*, or the indicated constructs were infiltrated into *N. benthamiana* leaves. The infiltrated leaves were photographed at 2 days post-inoculation. **C** and **D**, Expression levels of FoEPD1 and FgEPD1 in *N. benthamiana*. *N. benthamiana* leaves were infiltrated with *Agrobacterium* strains as indicated. Total protein was extracted for anti-FLAG western blotting.

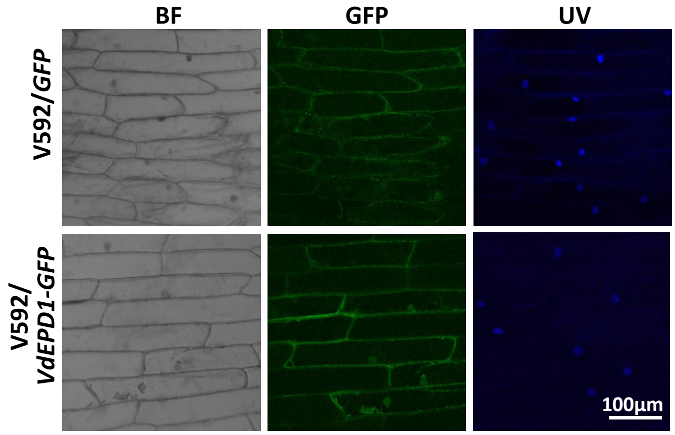

Figure S6. VdEPD1-GFP translocates into onion epidermal cells.
Conidia of the V592/*GFP* or V592/*VdEPD1-GFP* strain were inoculated onto onion epidermal cells. GFP and DAPI staining of nuclei (UV fluorescence) was visualized 3 days post-inoculation.

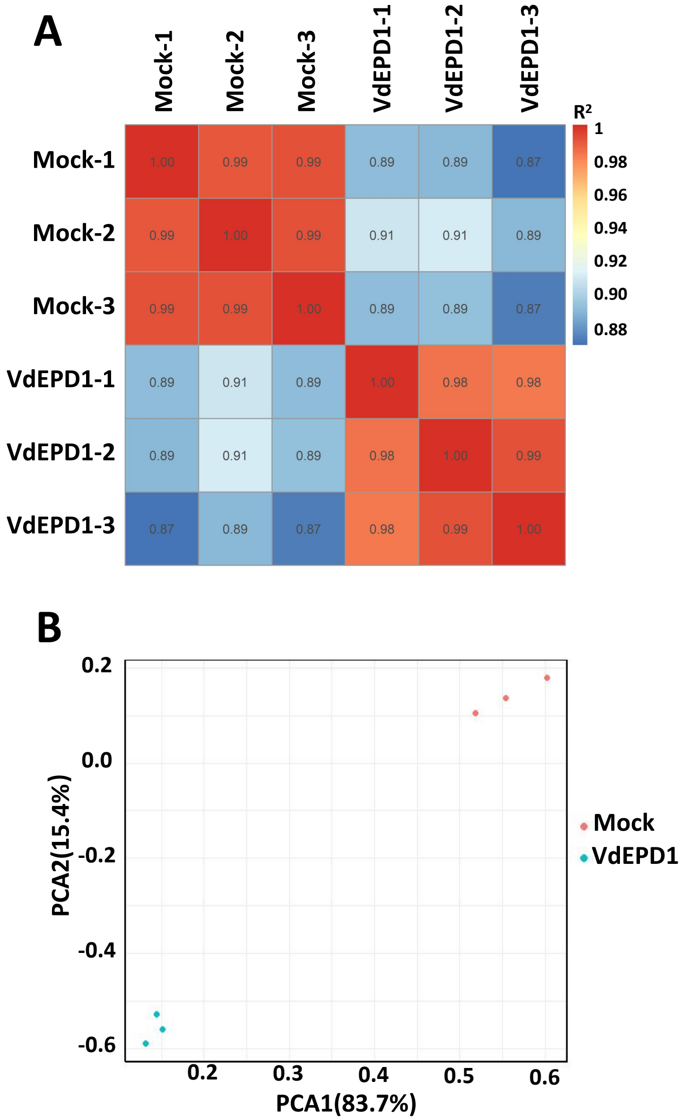

Figure S7. Data quality control analysis of the transcriptional changes triggered by VdEPD1 in *N. benthamiana*.

A, Pearson correlation coefficient matrix is used to quantify the similarity and correlation between transcriptome samples. The Mock group represents the buffer-treated samples, while the VdEPD1 group represents the samples with transient expression of VdEPD1. Three replicates are included in each group to ensure statistical robustness. The values at the intersection of the horizontal and vertical coordinates are the Pearson correlation coefficients (*r* values) between the transcriptome samples of the corresponding groups. **B**, Principal Component Analysis (PCA) visualizes the variation and clustering patterns among transcriptome samples based on their expression values measured in Fragments Per Kilobase of transcript per Million mapped reads. The horizontal and vertical coordinates represent different principal components, and the percentage represents the contribution of the corresponding principal component to the sample differences. Each point represents a sample, and the red points represent the three samples of the Mock group, while the green points represent the three samples with transient expression of VdEPD1.

A

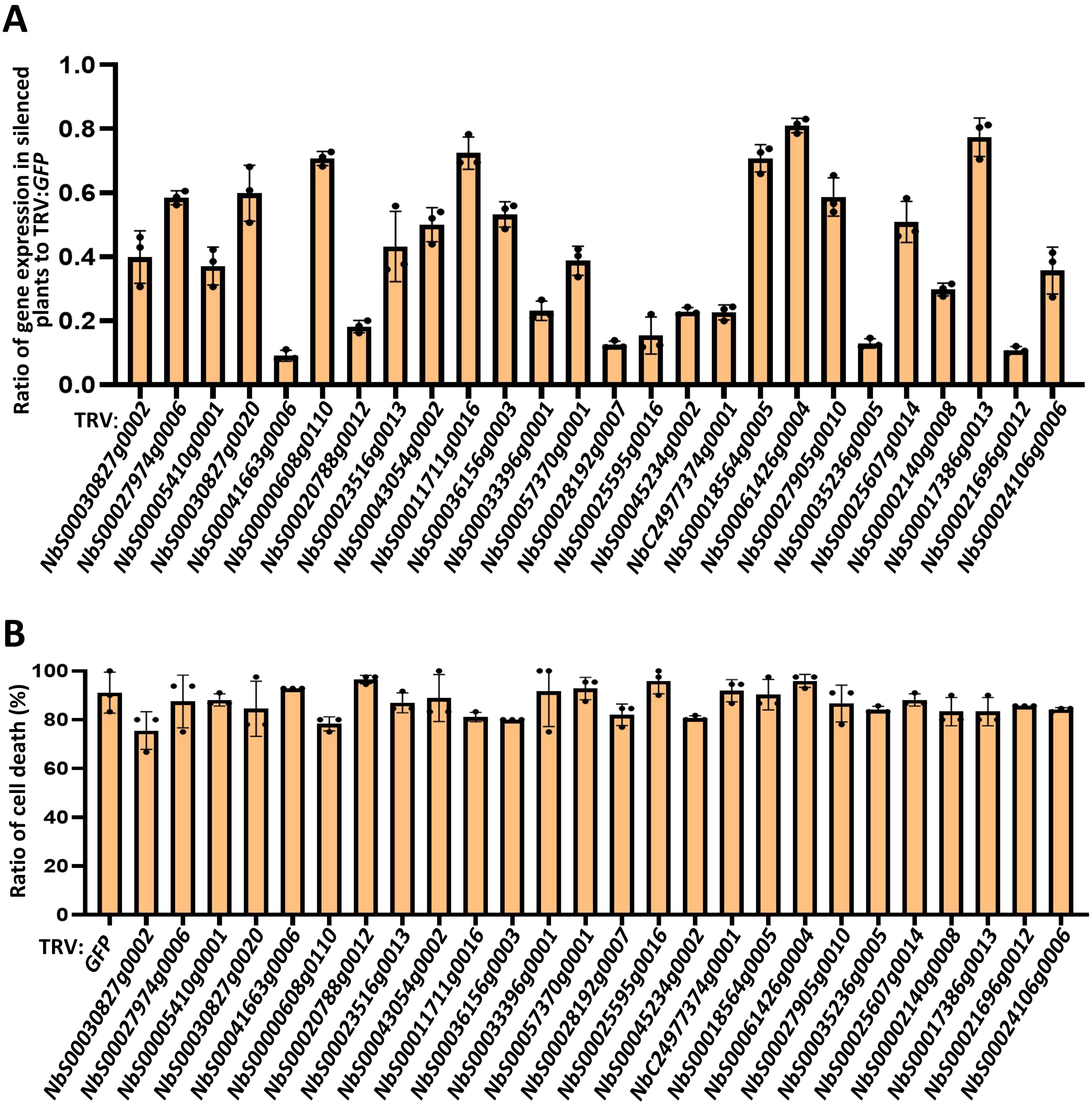

Figure S8. The indicated protein kinases-related encoding genes are not required for VdEPD1-induced cell death.

A, The expression levels of the protein kinase-related encoding genes are reduced in *N. benthamiana* plants. Total RNA from infiltrated plants was extracted for RT-qPCR analyses at 30 days post-inoculation. **B**, Silencing of the protein kinase-related encoding genes compromises VdEPD1-induced cell death. *Agrobacterium* carrying p*Cambia1300-35S-VdEPD1-FLAG*(OD_{600}: 0.8) was infiltrated into *N. benthamiana* plants where either the protein kinase-related encoding genes or the *GFP* gene was silenced. The percentage of cell death was recorded 3 days post-inoculation. Values are the means ± SD; *n* = 3. Error bars indicate standard deviation of 3 biological replicates.

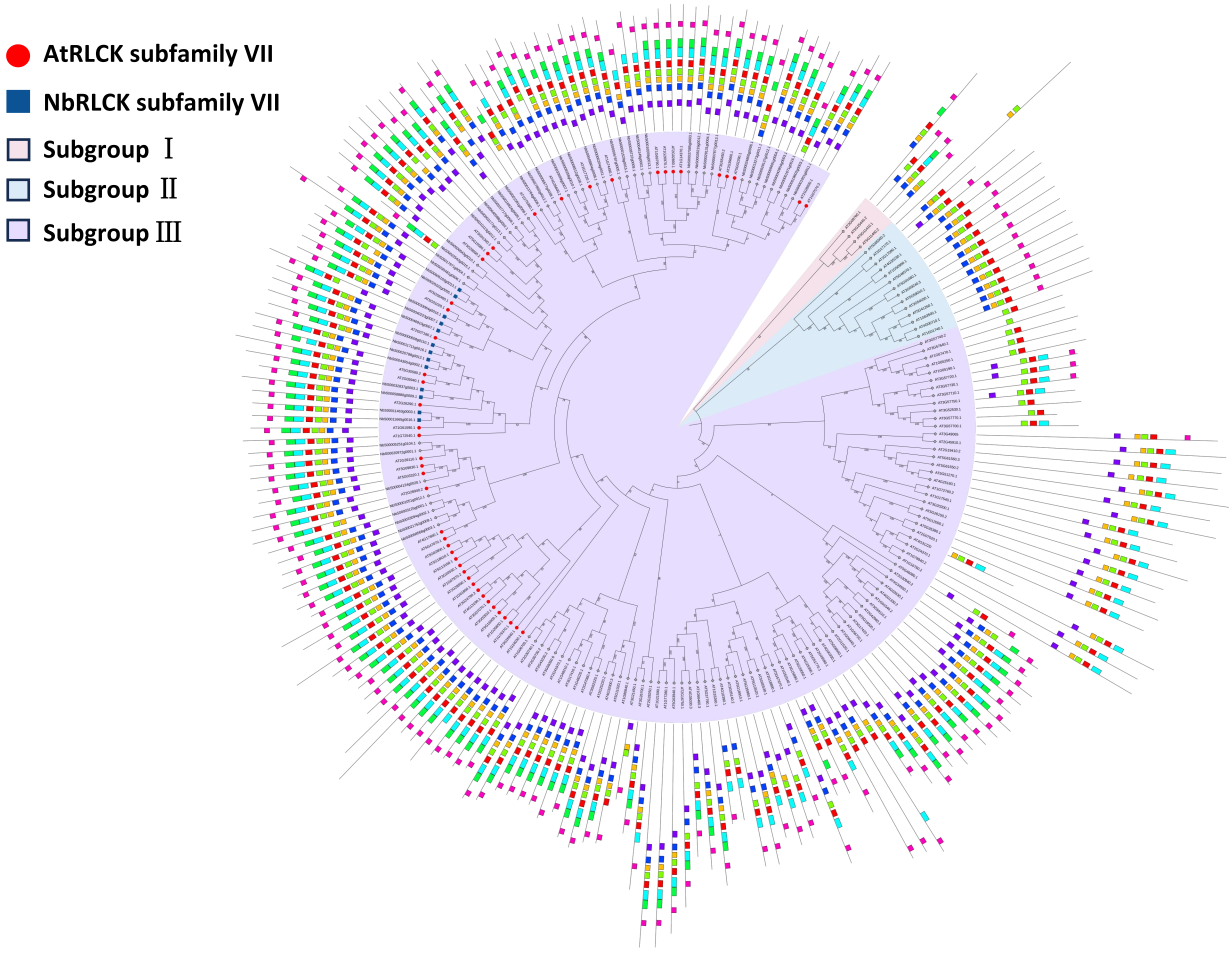

Figure S9. Phylogenetic analysis of the *Arabidopsis* receptor-like cytoplasmic kinases (RLCKs) and *N. benthamiana* RLCK subfamily VII members.

The phylogenetic tree was constructed based on an amino acid sequence alignment using the Neighbor-Joining method with p-distance and bootstrap analysis (1000 replicates). RLCKs from subfamily VII in *Arabidopsis* are highlighted with red triangles, while those from *N. benthamiana* are distinguished by blue squares. The tree includes a structural diagram for each RLCK, providing a detailed visual representation of their phylogenetic positions and structural features.

```
GbEIR1   --MTVMKITWRSIFPSCSKGVVKP--------ETKPKKEVTKQSSFNRLAMLELSYPST----MLTEDLSTSLAG-SNLHVFTLGELKVITQSFSSSNFLGEGGFG
GbEIR2A  MASLKRYLSLKNILPSCIKPKDS-----SSGPKIHATRQSPS----QRLSLTDVSHSG---SPLSLTDLSTSLIS---LHIFTLKELGVITHNFSKSNFLGEGGFG
GbEIR2D  MAALIRCLSLKNIIPSCIKPKDS-----SSGPKIHATRQSPS----QRLSLTDVSHSG---TPLSLNDLSTSLIS---LHIFTLKELGVITHNFSKANFLGEGGFG
GbEIR3A  -MIFKRYLSWKNILPNCIKPQVP-----SSGKKVHPSKRSPS----QRLSLSDISHFG---SPLSLNDLSNSLIS---LHVFTLKELELVTHKFSKSNFLGEGGFG
GbEIR3D  -MILKRYLSWKNILPNCIKPQVP-----SSGQKVHPSKRSPS----QRLSLSDISHFG---SPLSLNDLSNSLIS---LHVFTLKELELVTHKFSKSNFLGEGGFG
GbEIR4   -MTVKKKITWKSLMPSCYKRKDSS--------DSGENRLKLKPCQFQRISLSDVSDPSSP---ICVNDLSTSLFG-SNLYVFTLAELRLITHNFARCNLLGEGGFG
GbEIR5A  --MAVMKFTWRSIIPRCSKGIEEPE--AEAEAEPETKKQDSKQGSFSRLAMIDLSYPSS----RFTEDLSTSLAG-SNLYVFTLEELKVITQCFSSANFLGEGGFG
GbEIR5D  --MAVMKFTWRSIIPRCSKGIEE----AEAEAEAETKKQDSKQGSFSRLAMIDFSYPSS----MFTEDLSTSLAG-SNLYVFTLEELKVITQCFSSANFLGEGGFG
NbEIR    --MVASKIAWKFILPNCFKAKNDRNILPSETKIIQICKQINSDHHHSRLAISDISTDSR-SVFISLDDLSSNAIIGSNLHIFTYAELKIITSKFSSANFLGKGGFG

GbEIR1   PVHKGFIDDKLRPGLKAQPVAVKLLDLEGLQGHREWLTEVIFLAELRHPHLVKLIGYCCEEEHRLLVYEYMPRGSLENQLFRRYSVSLPWATRMKIALGAAKGLAY
GbEIR2A  PVYKGFIDDKFKPALKAQSVAVKVLDLDGTQGHREWLAEVIFLGQLKHPHLVNLIGYCYEEEHRLLVYEYMERGNLENQLFKRYGPPLPWLTRLKIALGTAKGLAF
GbEIR2D  PVYKGFIDDKFKPALKAQSVAVKVLDLDGTQGHREWLAEVIFLGQLKHPHLVNLIGYCYEEEHRLLVYEYMERGNLENQLFKRCGPPLPWLTRLKIALGTAKGLAF
GbEIR3A  PVYKGFIDDNLRPGLKAQSVAVKVLDLDGTQGHKEWLAEVILLGQLKHPHLVNLIGYCYEDEHRLLVYEYMERGSLENQLFKRYGPPLPWLTRLKIAVGTAKGLAF
GbEIR3D  PVYKGFIDDNLRPGLKAQSVAVKVLDLDGTQGHKEWLAEVILLGQLKHPHLVNLIGYCYEDEHRLLVYEYMERGSLENQLFKRYGPPLPWLTRLKIAVGTAKGLAF
GbEIR4   PVYKGFVDDKLRPGLKAQPVAVKTLDLDGLQGHREWLAEIIFLGQLRHPHLVKLIGYCYEDENRLLVYEYMPRGSLENQLFRRYSATLPWSTRMKIALGAAKGLAF
GbEIR5A  PVHKGFIDDNLRPGLEAQPVAVKLLDLEGLQGHREWLTEVVFLAQLSHPHLVKLIGYCCEDEHRLLVYEYMPRGSLENQLFAKYSVPLPWSTRMKIALGAAKGLAY
GbEIR5D  PVHKGFIDDNLRPGLEAQPVAVKLLDLEGLQGHREWLTEVVFLAQLSHPHLVKLIGYCCEDENRLLVYEYMPRGSLENQLFAKYSVPLPWSTRMKIALGAAKGLAY
NbEIR    PVHKGFIDDKIKPGLKAQPVAVKLLDLDGNQGHQEWLTEVVFLGQLRHPHLVKLIGYCWEEEQRLLVYEYMARGNLENHLFSRYSSCLPWSTRIKIAVGAAKGLAF

GbEIR1   LHEAEKPVIYRDFKASNILLDSDYNAKLSDFGLAKDGPEGDDTHVSTRVMGTQGYAAPEYIMTGHLTAMSDVYSFGVVLLELLTGRRSVDKNRCPREQNLVEWARP
GbEIR2A  LHEEEKPVIYRDFKTSNILLDLAYNAKVSDFGLATDGPEGEKSHVTTAVMGTEGYAAPEYITTGHLTTMSDVFSFGVVLLELLTGRRSVDKSRPAREKNLVEWARP
GbEIR2D  LHEEEKPVIYRDFKTSNILLDLAYNAKVSDFGLATDGPEGEKSHVTTAVMGTEGYAAPEYITTGHLTTMSDVFSFGVVLLELLTGRRSVDKSRPAREKNLVEWARP
GbEIR3A  LHEEEKPVIYRDFKTSNILLDSAYKAKVSDFGLATDGPAEEETHVTTCVMGTEGYAAPEYVSTGHLTTMSDVFSFGVVLLELLTGRRSVDKTRPSREKNLVEWARP
GbEIR3D  LHEEEKPVIYRDFKTSNILLDSAYKAKVSDFGLATDGPAEEETHVTTCVMGTEGYAAPEYVSTGHLTTMSDVFSFGVVLLELLTGRRSVDKTRPSREKNLVEWARP
GbEIR4   LHEADKPVIYRDFKSSNILLDSDYNCKLSDFGLAKDGPEGGETHVTTRVMGTQGYAAPEYIMTGHLTTMSDVYSFGVVLLELLTGKRSMDNTRPSREQSLVEWARP
GbEIR5A  LHEAEKPVIYRDFKASNILLDSDYSAKLSDFGLAKDGPEGDKTHVSTRVMGTRGYAAPEYIMTGHLTAMSDVYSFGVVLLELLTGRRSLDKSRSPREQNLAEWARP
GbEIR5D  LHEAEKPVIYRDFKASNILLDSDYSAKLSDFGLAKDGPEGDKTHVSTRVMGTRGYAAPEYIMTGHLTAMSDVYSFGVVLLELLTGRRSLDKSRSPREQNLAEWARP
NbEIR    LHGEEKPVIYRDFKASNILLDSDYKAKLSDFGLAKDGPEGDDTHVSTRVMGTHGYAAPEYIMTGHLTSKSDVYSFGVVLLELITGRPAMDKNRPIRERNLVDWARP

GbEIR1   MLNDARKLGRIMDPRLEGQYSETGARKAAALAYQCLSHRPKQRPKMSDVVKTLEPLQDYEDVLVGPFVYTVPTQSDKPKQ-DEDTVTKECEPKKEK----------
GbEIR2A  SLKDPYKLDAIMDPRLEGQYSTEGVKKVAALASQCLSNHPKSRPTMSNVVKALEPLLDLTDIPTGPFVYIVPTDGNSEPIIQNIIQREEKQHDELRIEKNIDRKEE
GbEIR2D  SLKDPYKLDAIMDPRLEGQYSTAGVKKVAALAYQCLSNHPKSRPTMSNVVKALESLLDLTDIPTGPFVYIVPTNGNSEPIIQNIIHREEKQHDELRIEKNIDHKEE
GbEIR3A  LLKDPHKLHGIMDPRLEGQYSTEGARKAAALAYQCLSSHPKSRPTMRNVVKGLQPLLHLTDIPIGPFVYIAPTQENKEPTVQKIEEIYEKQFDEVKMEK--------
GbEIR3D  LLKDPHKLHGIMDPRIEGQYSTEGARKAAALAYQCLSSHPKSRPTMRNVVKGLQPLLHLTDIPIGPFVYIAPTQDNKEPTVQKIEEIYEKQFDEVKIEK--------
GbEIR4   LLRDPKRLDRVIDPRLEGQYSNKGAQKVAALAYKCLSHYPKPRPTMGDVVIVLESVQGFEDEFVGPFVYVVP---------NEADDSNEFLAKKGE----------
GbEIR5A  MLNESRRLARIMDPKLEGQYSETGARKAAALAYQCLSHRAKQRPKMSDVVNILEPLLDYEETSVGTFVYTVPTHQNGGSPPKDDTDTKECEAKTELKKENGDHH--
GbEIR5D  MLNESRRLARIMDPKLEGQYSETGARKAAALAYQCLSHRAKQRPKMSDVVNILEPLLDYGETSVASFVYTVPTHQKGGSPPKDDTDTKECEAKTELKKENGHHR--
NbEIR    MLRDFHKLDRIMDPRLEGQYSTEGAKKVAALAYQCLSHQPRSRPTMSNVVKSLEPISELTDIPIGPFVYVVPS--SECDKELEIGALKSKLDEEKKMNVN------

GbEIR1   -CHHHENHKHHHRHHRHQTRS-PRMSAIHSERDALKRNHRNGLNSPLHFKVREAA
GbEIR2A  KKEKNRLPHRKARKHRPRVKP-SRSRAVYSDTDLYKVLGSSLYTPKH--------
GbEIR2D  KKEKNRLPHRKARKHRPRVKP-SRSRAVYSDTDLYKVLGSSLYTPKH--------
GbEIR3A  KEDKSRIPRRKGRKHRRRVKP-SRSRAMYSDTDLYRTLGSSLYSPKH--------
GbEIR3D  KEDKSRIPRRKGRKHRRRVKP-SRSRAMYSDTDLYRSLGSSLYSTKH--------
GbEIR4   ----NE-HDSPLRGWRNRIKL-PPSSVANAESPCSI-------------------
GbEIR5A  NRHHHRRSHKSRDGHRHRNKS-SSQSSVHSENYTSKQTLENGSNEECNID-----
GbEIR5D  NRHHHRRSHKSRDGHRHQNKS-SSQSSVHSENDTSKQNLENGSNEECNID-----
NbEIR    -EDEQKGARRHGHRHKHRPKSSAAAAAVYSDTHMQNFALSHERTNKKHILK----
```

Figure S10. An alignment of NbEIR homologous proteins in *G. barbadense* plants. NbEIR homologous proteins in *G. barbadense* were aligned using Vector NTI software.

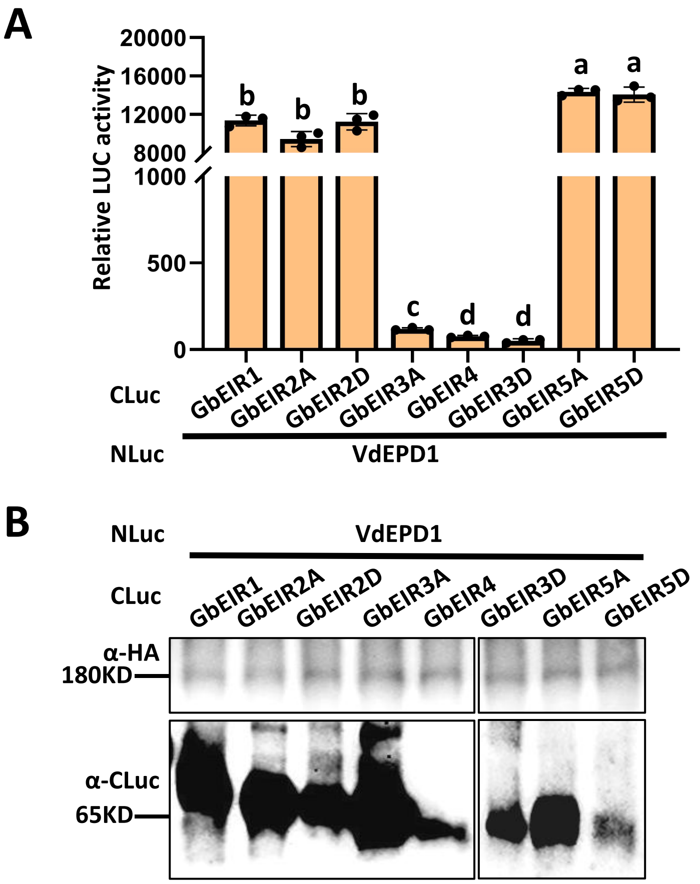

Figure S11. GbEIR1, GbEIR2A, GbEIR2D, GbEIR5A and GbEIR5D interact with VdEPD1.

A, VdEPD1 interacts with GbEIR1, GbEIR2A, GbEIR2D, GbEIR5A, and GbEIR5D in *N. benthamiana*. *N. benthamiana* leaves infiltrated with the indicated constructs were sliced into strips, and their relative luminescence was determined using a microplate luminometer. Values are the means ± SD; *n* = 3. Error bars indicate standard deviation of 3 biological replicates. Different letters indicate significant differences, as determined by one-way ANOVA. **B**, All genes encoding NLuc proteins were further fused with a 3×HA tag in the NLuc-vector. Anti-HA and anti-CLuc immunoblots were used to detect the levels of NLuc-fusion and CLuc-fusion proteins, respectively.

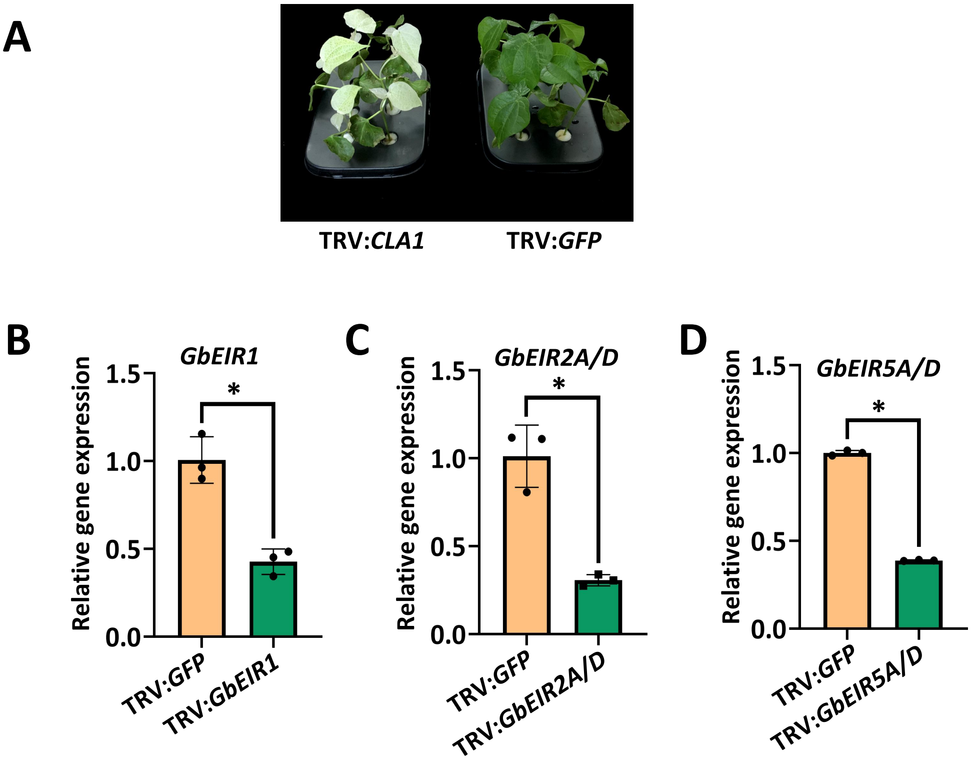

Figure S12. Verification of virus-induced gene silencing in *G. barbadense*.

A, Preliminary assay of the efficiency of VIGS under our experimental conditions. Ten-day-old cotton plants were infiltrated with *Agrobacterium* carrying VIGS-control vector (*TRV:GFP*) and *TRV:CLA1*. The photographs were taken 2 weeks after infiltration. **B**-**D**, The expression levels of *GbEIR1* (**B**), *GbEIR2A/D* (**C**), *GbEIR5A/D* (**D**) are reduced in cotton plants. Total RNA was extracted from plants infiltrated with *Agrobacterium* carrying *pTRV1* together with *pTRV2-GbEIR1*, *pTRV2-GbEIR2A/D*, and *pTRV2-GbEIR5A/D* plants for RT-qPCR analyses of *GbEIR1*, *GbEIR2A/D*, and *GbEIR5A/D* expression. Values are the means ± SD; n = 3. Error bars indicate standard deviation of 3 biological replicates. Student's t-test was carried out to determine the significance of difference. *Indicates significant difference at a p-value of < 0.05.

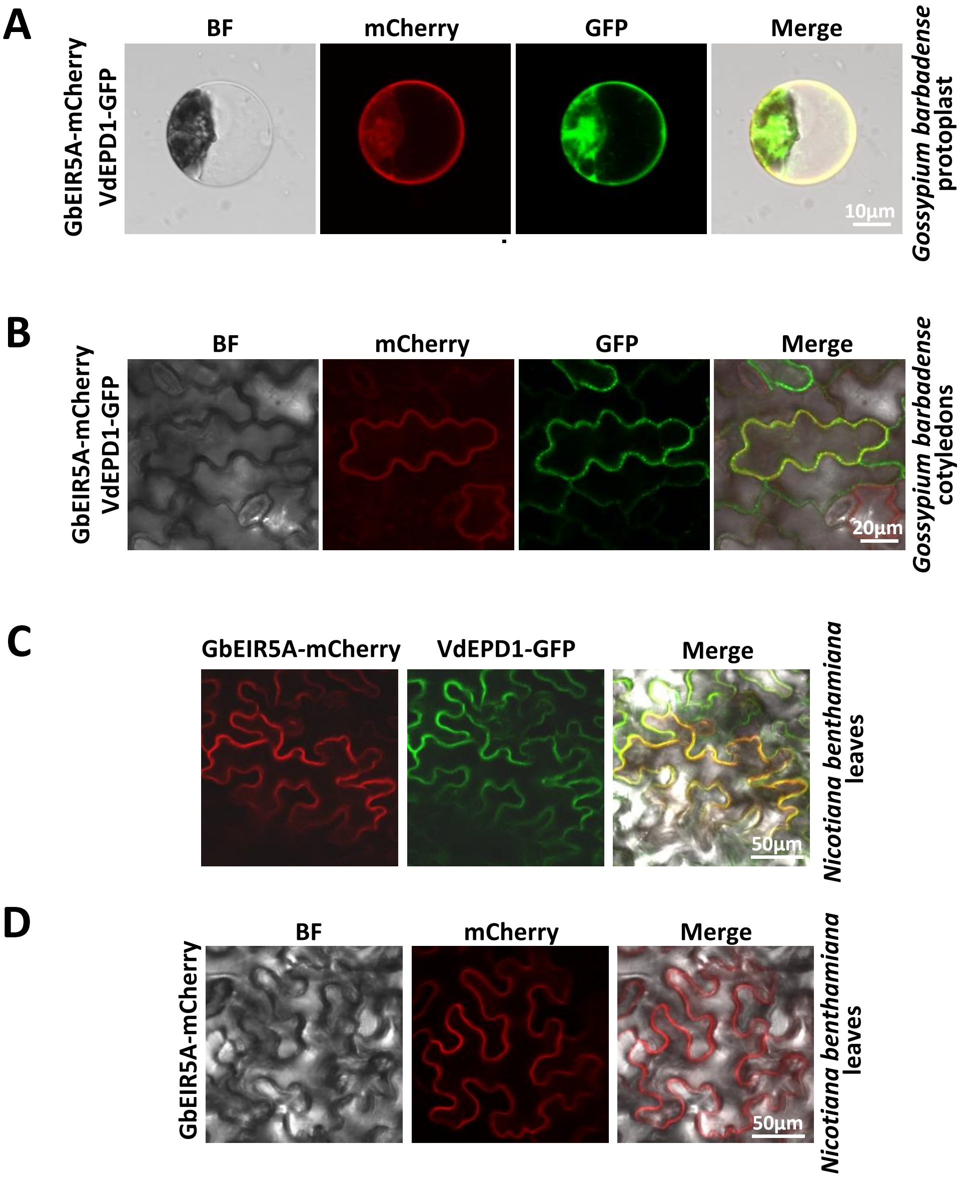

Figure S13. VdEPD1 co-localizes with GbEIR5A.
A-**C**, VdEPD1-GFP and GbEIR5A-mCherry co-localize in *G. barbadense* protoplasts (**A**), *G. barbadense* leaves (**B**), and *N. benthamiana* leaves (**C**). *G. barbadense* protoplasts were co-transfected with VdEPD1-GFP and GbEIR5A-mCherry plasmids as indicated. *G. barbadense* or *N. benthamiana* leaves were infiltrated with *Agrobacterium* strains carrying *VdEPD1-GFP* and *GbEIR5A-mCherry* plasmids as indicated. GFP and mCherry fluorescence were visualized. **D**, Localization of GbEIR5A-mCherry in *N. benthamiana* leaves. *N. benthamiana* leaves were infiltrated with *Agrobacterium* strains carrying GbEIR5A-mCherry plasmids as indicated. mCherry fluorescence was visualized.

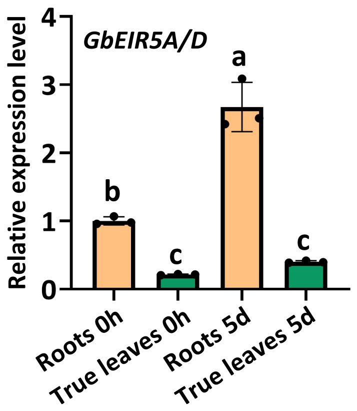

Figure S14. Expression levels of *GbEIR5A/D* in the roots and true leaves of *G. barbadense*.

Total RNA was extracted from the roots and true leaves of uninfected *G. barbadense* and from *G. barbadense* infected with *V. dahliae* for 5 days. RT-qPCR analysis was performed to assess the expression levels of *GbEIR5A/D* in the root and true leaf tissues of *G. barbadense*. Values are the means ± SD; *n* = 3. Error bars indicate standard deviation of 3 biological replicates. Different letters indicate significant differences, as determined by one-way ANOVA.

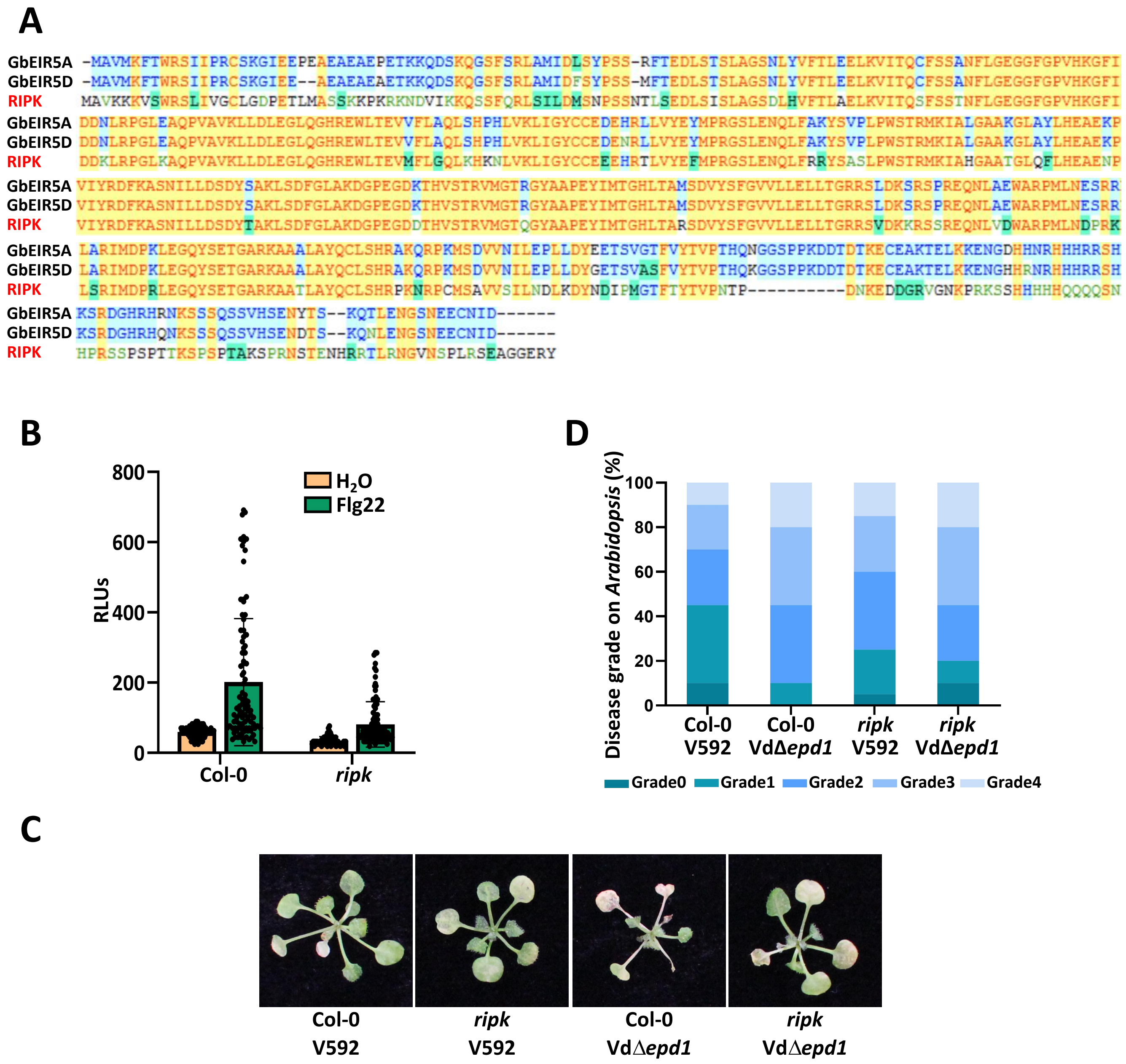

Figure S15. The *ripk* mutant showed reduced levels of flg22-induced ROS in comparison to wild-type (Col-0) plants.

A, An alignment of GbEIR5A, GbEIR5D, and RIPK using Vector NTI software. **B**, *ripk* mutant plants exhibit a compromised flg22-induced oxidative burst. Leaf strips from *ripk* mutant or Col-0 plants were incubated in H_2O overnight. 1 μM flg22 was added, and the production of ROS was immediately measured using a luminol-based assay. Relative amounts of H_2O_2 are shown as relative luminescence units. **C** and **D**, RIPK is required for VdEPD1-mediated defense in *Arabidopsis*. Disease symptoms (**C**) and index analyses (**D**) of *ripk* mutant and Col-0 plants infected with V592 and VdΔ*epd1* mutant strains.Three-week-old *Arabidopsis* seedlings were inoculated with V592 or VdΔ*epd1*. Plants were photographed, and disease index analyses 3-4 weeks post-inoculation. Disease indexes were evaluated with 3 replicates generated from 24 plants (*n* = 24) for each inoculum. The experiment was repeated 3 times with similar results.
